# Beyond the Simple Copper(II) Coordination Chemistry with Quinaldinate and Secondary Amines

**DOI:** 10.3390/molecules25071573

**Published:** 2020-03-30

**Authors:** Barbara Modec, Nina Podjed, Nina Lah

**Affiliations:** Department of Chemistry and Chemical Technology, University of Ljubljana, Večna pot 113, 1000 Ljubljana, Slovenia

**Keywords:** copper(II) complexes, quinaldinate, secondary amines, pyrrolidine, morpholine, piperidine, reduction, crystal structure

## Abstract

Copper(II) acetate has reacted in methanol with quinaldinic acid (quinoline-2-carboxylic acid) to form [Cu(quin)_2_(CH_3_OH)]∙CH_3_OH (**1**) (quin^−^ = an anionic form of the acid) with quinaldinates bound in a bidentate chelating manner. In the air, complex **1** gives off methanol and binds water. The conversion was monitored by IR spectroscopy. The aqua complex has shown a facile substitution chemistry with alicyclic secondary amines, pyrrolidine (pyro), and morpholine (morph). *trans*-[Cu(quin)_2_(pyro)_2_] (**2**) and *trans*-[Cu(quin)_2_(morph)_2_] (**4**) were obtained in good yields. The morpholine system has produced a by-product, *trans*-[Cu(en)_2_(H_2_O)_2_](morphCOO)_2_ (**5**) (morphCOO^−^ = morphylcarbamate), a result of the copper(II) quinaldinate reaction with ethylenediamine (en), an inherent impurity in morpholine, and the amine reaction with carbon dioxide. (pyroH)[Cu(quin)_2_Cl] (**3**) forms on the recrystallization of [Cu(quin)_2_(pyro)_2_] from dichloromethane, confirming a reaction between amine and the solvent. Similarly, a homologous amine, piperidine (pipe), and dichloromethane produced (pipeH)[Cu(quin)_2_Cl] (**11**). The piperidine system has afforded both mono- and bis-amine complexes, [Cu(quin)_2_(pipe)] (**6**) and *trans*-[Cu(quin)_2_(pipe)_2_] (**7**). The latter also exists in solvated forms, [Cu(quin)_2_(pipe)_2_]∙CH_3_CN (**8**) and [Cu(quin)_2_(pipe)_2_]∙CH_3_CH_2_CN (**9**). Interestingly, only the piperidine system has experienced a reduction of copper(II). The involvement of amine in the reduction was undoubtedly confirmed by identification of a polycyclic piperidine compound **10**, 6,13-di(piperidin-1-yl)dodecahydro-2*H*,6*H*-7,14-methanodipyrido[1,2-*a*:1′,2′-*e*][1,5]diazocine.

## 1. Introduction

The coordination chemistry of copper is very rich because of its biological roles [1,2,3] and diverse practical applications, e.g., as catalysts, fungicides, and pesticides [4]. The chemistry of no other transition metal surpasses that of divalent copper with *N*- and *O*-donor ligands [4]. The reactivity of copper in its metalloenzymes and proteins rests mostly in its redox-active character, as they are involved in electron transfer, oxygen transport, and oxidation of important substrates such as amines, L-ascorbic acid, galactose, etc. [4,5]. Under oxidizing conditions in the cell, copper exists as Cu^2+^, whereas reducing conditions favor the Cu^+^ form. Different behavior of the two states may be traced to differences in their hardness: whereas the Cu^2+^ ion is classified as a borderline Lewis acid, the reduced counterpart, the Cu^+^ ion, is an exemplary soft acid [6]. Hard *N*- and *O*-donors dominate the coordination chemistry of divalent state, whereas the Cu^+^ ion favors ligands with soft donor atoms, such as phosphorus, sulfur, or iodine. Because of the spherical symmetric *d*^10^ configuration, the Cu^+^ ion lacks any LFSE and it has, as a consequence, no preference for a specific coordination environment [5]. On the opposite, the Cu^2+^ ion usually displays a distorted octahedral environment with four tightly bound donors in a plane and two occupying more distant sites above and below this plane. In the limit, the elongation of the axial bonds often results in a square-planar geometry [5]. The described distortions of the coordination polyhedra are due to the operating Jahn–Teller effect, a characteristic of a metal complex with the *d*^9^ electron configuration [7]. The ligands and their spatial distribution can induce changes in the reduction potential of the metal ion and thereby influence its oxidation state [8]. The research on the Cu^2+^ and Cu^+^ model systems with a common ligand environment can give information about the metal’s mode of action at the active sites in enzymes [8].

**Scheme 1:**
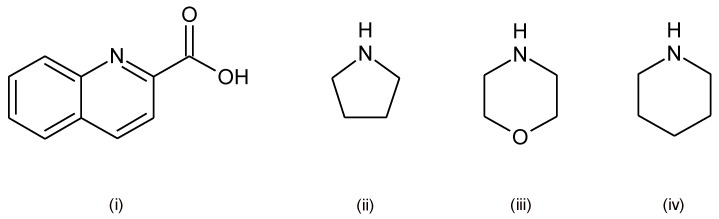
Structural formulae of the ligands used in this work: (i) Quinaldinic acid, (ii) pyrrolidine, (iii) morpholine, and (iv) piperidine.

Our current study involves copper(II) complexes with quinaldinate and alicyclic secondary amines as auxiliary ligands. Quinaldinic acid, with a rational name quinoline-2-carboxylic acid (shown in Scheme 1), is a biological molecule, mostly known for its role in tryptophan metabolism [9]. Its anionic form, abbreviated as quin^−^, readily forms complexes with many transition metal ions and has, therefore, found use in their quantitative gravimetric determination [10,11]. Structurally characterized complexes with quinaldinate reveal a bidentate chelating manner through pyridine nitrogen and carboxylate oxygen as a prevailing coordination mode [12,13,14,15,16,17,18,19,20,21,22]. Examples of a bridging mode through two or all three donor atoms are not very common [23,24,25,26]. The quinaldinate was introduced into our reaction system through the [Cu(quin)_2_(H_2_O)] starting material, one of the rare copper(II) quinaldinate compounds known prior to this report [27]. The choice of auxiliary ligands, pyrrolidine, morpholine, and piperidine (Scheme 1), was governed by their ability to bind uniformly in a monodentate manner. The group shares a highly basic character and complete miscibility with a large number of solvents. Besides, their NH moiety makes them good hydrogen bond donors. Such an ensemble of ligands is of interest also from the viewpoint of crystal engineering and chemical recognition.

Herein, we present products of the [Cu(quin)_2_(H_2_O)] reactions with the selected amines. The compounds were characterized by X-ray structure analysis on a single crystal and infrared vibrational spectroscopy. A series of novel compounds may be divided into two groups. The first one is comprised of the desired amine complexes, [Cu(quin)_2_(pyro)_2_] (**2**), [Cu(quin)_2_(morph)_2_] (**4**), [Cu(quin)_2_(pipe)] (**6**), [Cu(quin)_2_(pipe)_2_] (**7**), [Cu(quin)_2_(pipe)_2_]∙CH_3_CN (**8**), and [Cu(quin)_2_(pipe)_2_]∙CH_3_CH_2_CN (**9**). The second group, where products of several unexpected reactions were assembled, illustrated both the reactivity of copper(II) and, as a consequence, the unpredictability of the reaction outcome. [Cu(quin)_2_Cl]^−^, a complex with chloride, was obtained as a pyrrolidinium (**3**) or a piperidinium (**11**) salt, when the corresponding amine reacted with dichloromethane, used as a solvent. *trans*-[Cu(en)_2_(H_2_O)_2_](morphCOO)_2_ (**5**) (en = ethylenediamine) was a result of the quinaldinate displacement with the morpholine impurity, ethylenediamine. The counter-anion of **5** was product of yet another inadvertent reaction: the one between morpholine and carbon dioxide. The piperidine reaction system presents itself as the most enigmatic of all. It yielded, apart from three metal complexes, a polycyclic piperidine compound **10,** which was a product of a complicated electron transfer between amine and Cu^2+^. It is to be noted that copper(II)-assisted transformations of organic substrates often accompany the coordination chemistry of this metal ion [28,29,30,31,32,33,34,35]. Our study provides the basis for the ongoing work on the reduction of copper(II) with piperidine and its homologs.

## 2. Results and Discussion

### 2.1. Synthetic Considerations

Based on the previous study of related zinc(II) complexes with quinaldinate [36], [Cu(quin)_2_(H_2_O)] was expected to undergo a straightforward substitution of water with the amine ligand. At fairly mild conditions, the displacement reactions should result in complexes with one or two amine ligands, [Cu(quin)_2_(amine)] and [Cu(quin)_2_(amine)_2_]. With the latter composition, two geometric isomers are possible. To our surprise, the behavior of the three chosen amines, in spite of their high likeness, was profoundly different. Unless stated otherwise, the described reactions were carried out at ambient conditions.

In the case of morpholine, a virtually insoluble [Cu(quin)_2_(morph)_2_] (**4**) with a *trans* disposition of ligands was the first isolated solid. The violet color of the second crystalline phase ruled out its composition to be any of the desired heteroleptic copper(II) complexes with quinaldinate and amine. Namely, complexes containing these two ligands are typically blue to green. Results of the X-ray structure analysis took us by surprise as the compound was identified as *trans*-[Cu(en)_2_(H_2_O)_2_](morphCOO)_2_ (**5**). Two of its components called for further explanations. The first one was morphylcarbamate, a counter-anion, which forms upon the morpholine reaction with carbon dioxide. This reaction was hardly without precedence [37,38,39,40,41].

morph + CO_2_ → morphCOOH

morphCOOH + morph → morphH^+^ + morphCOO^−^

The first product, 1-morpholinecarboxylic acid, reacts with the excess of morpholine and a salt is formed. Owing to the rich electron density on oxygen atoms, the carbamate ion was reported to be unstable. It could attain stability with the delocalization of electrons through hydrogen bonds [40]. The same mechanism is apparently at work in the case of [Cu(en)_2_(H_2_O)_2_](morphCOO)_2_ (**5**), where the carboxylate moiety participates in hydrogen bonding interactions. The other, a more intriguing component of **5**, is the ligand ethylenediamine. The most imminent question concerns its source. Ethylenediamine was identified as a common impurity in morpholine, with its content in the 0.006–0.081% *w*/*w* range, depending upon the supplier [42]. In a control experiment, when we used a recently acquired morpholine, our results were reproduced. The filtrate, which gave single crystals of both [Cu(quin)_2_(morph)_2_] (**4**) and *trans*-[Cu(en)_2_(H_2_O)_2_](morphCOO)_2_ (**5**), contained a very small amount of copper(II). The amount of ethylenediamine was apparently large enough to replace the bound quinaldinates. The end result of the competition between the two chelating ligands provides yet another evidence of a huge affinity of copper(II) towards ethylenediamine, which is distinguished by its high conformational flexibility, a property that quinaldinate lacks.

The pyrrolidine reaction system behaved in a predicted manner as it produced a *trans* isomer of [Cu(quin)_2_(pyro)_2_] in good yield. The crystalline solid, labelled **2**, is poorly soluble in the majority of solvents with the exception of dichloromethane and chloroform. Its recrystallization from dichloromethane inadvertently resulted in (pyroH)[Cu(quin)_2_Cl] (**3**), a product, which confirms that pyrrolidine, initially present as a ligand in the copper(II) complex, reacted with the solvent. With dichloromethane, commonly employed as a solvent in synthesis and extraction processes, undesired reactions with primary and secondary aliphatic amines were observed previously, in particular when the solutions were left to stand for extended periods [43,44,45]. Later studies have confirmed that pyrrolidine rapidly reacts with dichloromethane at room temperature with the major products being 1,1′-methylenebis(pyrrolidine), known as aminal, and pyrrolidinium hydrochloride. Although aminal was not isolated in our case, its formation finds ample evidence in the literature [43]. The in situ formed chloride, which has many times proved to be competitive with other monodentate ligands, coordinated to copper(II). Piperidine was expected to react in an analogous manner [46]. However, the reaction that yielded (pipeH)[Cu(quin)_2_Cl] (**11**) differs from the one that afforded **3**. Dichloromethane was added directly to the reaction mixture that normally produces copper(II) quinaldinate complexes with piperidine (please see the Materials and Methods). Yet, the reason for its introduction lies in the in situ formation of a sufficient amount of chloride that would allow its coordination to copper(I), a reduced form of the metal, and hopefully, crystallization of the cuprous complex. As will be shown presently, the piperidine/acetonitrile/Cu(II) mixture experiences a reduction of metal ions. Instead, a small number of crystals of (pipeH)[Cu(quin)_2_Cl] (**11**) was isolated from an orange-yellow solution that retained its color when exposed to the air. The color and its stability speak of the presence of the tetrahedral [CuCl_4_]^2−^, which can be of an orange color [4,47]. A huge excess of chloride makes a complete substitution in the copper(II) coordination sphere a highly probable process. Unfortunately, the presence of the [CuCl_4_]^2−^ ions introduces an ambiguity about the copper(II) reduction. Namely, the colors of the reduced solution and of the one containing the tetrahedral [CuCl_4_]^2−^ ions are very similar. Irrespective of the actual situation, our goal, the isolation of copper(I) complex, was not achieved by using this synthetic strategy.

**Scheme 2:**
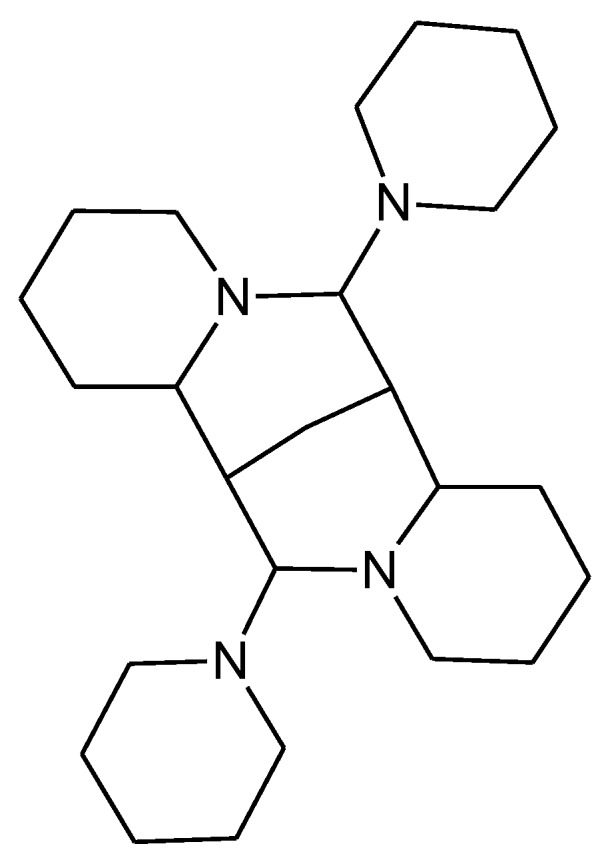
Structural formula of 6,13-di(piperidin-1-yl)dodecahydro-2*H*,6*H*-7,14-methanodipyrido[1,2-*a*:1′,2′-*e*][1,5]diazocine, polycyclic piperidine derivative (**10**).

The prototypic reaction of copper(II) starting material with piperidine in acetonitrile requires further discussion. Within a few minutes after the complete consumption of [Cu(quin)_2_(H_2_O)], a blue solid, identified as [Cu(quin)_2_(pipe)] (**6**), started to precipitate. If no care is exercised, the transient mono-substituted complex reacts further with amine to *trans*-[Cu(quin)_2_(pipe)_2_], which crystallizes in two forms, in a non-solvated one or with acetonitrile solvent molecules. Pure [Cu(quin)_2_(pipe)_2_] (**7**), which has a more condensed structure, as compared to the channel-like structure of [Cu(quin)_2_(pipe)_2_]∙CH_3_CN (**8**), can be obtained reproducibly at solvothermal conditions. The result is in line with the expectation that more forcing conditions afford a denser structure with a lower level of solvation [48]. The most remarkable characteristic of the piperidine system is a change of color from green to deep red-brown that sets in after 3 to 4 days of stirring. On exposure to the air, the color promptly changed to green. The first change can be explained by the reduction of Cu^2+^ to Cu^+^, and the second by the Cu^+^ re-oxidation with the elemental oxygen. Our attempts at the isolation of the reduced metal species were not met with success. Apart from the addition of dichloromethane (see above), the concentration of the copper starting material was also increased. The only solid that precipitated from the red-brown solution, kept in a closed container, was a mixture of crystalline [Cu(quin)_2_(pipe)_2_] (**7**) and [Cu(quin)_2_(pipe)_2_]∙CH_3_CN (**8**). In another attempt, a different starting material, CuCl_2_∙2H_2_O, was used. Although the change allowed isolation of a highly crystalline mono-piperidine complex **6**, the behavior of a modified system was essentially the same. Another notable feature of the piperidine system is that it acquires a distinct odor of ammonia gas. In one instance only, colorless crystals of **10** (Scheme 2) grew from a reaction mixture that was kept at 5 °C for 2 months. Compound **10** lacks a complete characterization as it was not available in pure form, and a reproducible bulk synthesis was not achieved. Its true identity was shown by the X-ray diffraction analysis on a single crystal. The polycyclic compound **10** consists of four whole piperidine rings, fused together with five methine/methylene carbon atoms. Compound **10** gives an undisputed proof of piperidine involvement in the one-electron reduction of copper(II). The reduction of Cu^2+^ with piperidine in acetonitrile has been reported previously [49]. An electron transfer from nitrogen lone pair to Cu^2+^ ion was confirmed by ESR spectroscopy as the initial step in the reaction [50], yet at the time, a detailed knowledge of the free radical formed could not be drawn. Interestingly, no free radicals could be detected in a similar reaction of pyrrolidine. The amine radical cations, which form upon the electron loss from the parent amine, are known to display several modes of reactivity, which include C–C cleavage, hydrogen atom abstraction, the formation of reactive iminium ions, etc. [51]. The structure of **10** implies that a series of reactions with some involving the radical species was at work. Their complexity could be an answer as to why we did not succeed, after numerous attempts, at repeating the preparation of **10**.

An important question also pertains to the nature of the reduced metal species. Failure at its isolation suggests that our system lacks ligands that stabilize the reduced state. The Cu^+^ ion is known to prefer soft ligands such as phosphines or iodide [6]. Furthermore, complexes with saturated *N*-donor ligands, as exemplified by piperidine, are generally less stable than the ones with unsaturated/aromatic ligands [4]. Contrary to the expectations, the literature reveals several copper(I) complexes with piperidine and none with quinaldinate, as demonstrated by [Cu(pipe)_2_Cl] [52], [{Cu(PPh_3_)(pipe)X}_2_] (X^−^ = halide) [53] and [Cu_4_(pipe)_4_I_4_] [54,55]. Pertinent to our discussion is a dark red compound with polymeric [Cu_2_I_3_]^−^ ions, which crystallized with the {(Hquin)_2_H}^+^ counter-cations [56]. The acidic medium during its synthesis prevented the formation of quinaldinate and its subsequent coordination to copper(I). Its color was explained in terms of a charge-transfer electronic transition tailing into the visible region.

The role of the solvent, acetonitrile, in the reduction, should not be overlooked. Acetonitrile has been reported to effectively solvate the Cu^+^ ion, thereby making it more stable towards disproportionation or oxidation with oxygen [57]. It should be emphasized that our pyrrolidine or morpholine reaction mixtures did not show color changes that would suggest a reduction of metal ions.

### 2.2. Solid State Structures

**Figure 1:**
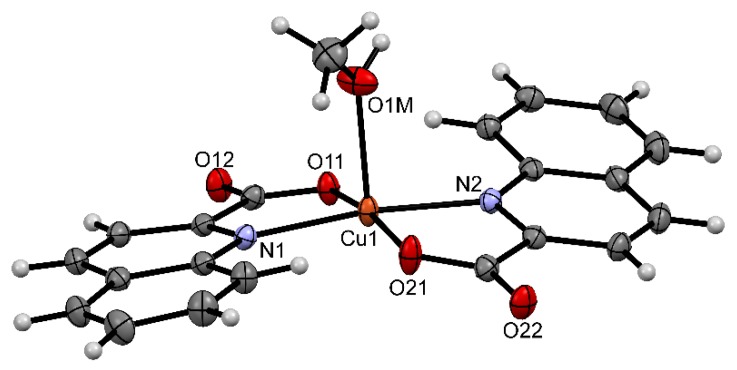
ORTEP drawing of [Cu(quin)_2_(CH_3_OH)], a complex molecule of **1**. Displacement ellipsoids are drawn at the 50% probability level. Hydrogen atoms are shown as spheres of arbitrary radii.

Relevant structural features of the novel compounds are given first, whereas comparison with related literature examples is at the end of this section. The methanol complex, [Cu(quin)_2_(CH_3_OH)], crystallizes with one solvent molecule of methanol per formula unit. Solvent molecules render the turquoise crystals of [Cu(quin)_2_(CH_3_OH)]∙CH_3_OH (**1**) unstable: crystals lose their luster rapidly when not in contact with the mother liquor. The copper(II) ion of [Cu(quin)_2_(CH_3_OH)] features a five-coordinate environment that consists of two bidentate *N*,*O*-chelating quinaldinates and a methanol molecule (Figure 1). With a chelating coordination of quinaldinate, a five-membered metallacycle is formed. The N_2_O_3_ donor set defines vertices of a square pyramid with the quinaldinate donors in its basal plane and the methanol oxygen occupying its axial site. The copper(II) ion is lifted ca. 0.17 Å above the basal plane towards the methanol oxygen. The *τ* parameter value, 0.05, agrees well with the square-pyramidal environment [58]. As shown in Figure 1, the relative disposition of quinaldinates is *trans*. A dihedral angle of 21.01(3)° is formed between the best planes of quinaldinates. The longest bond, i.e., the copper-to-methanol bond with the value of 2.2806(14) Å, is a result of the Jahn–Teller effect. It is slightly shorter from 2.33 Å, the average value observed for the Cu–alcohol bonds [59].

**Figure 2:**
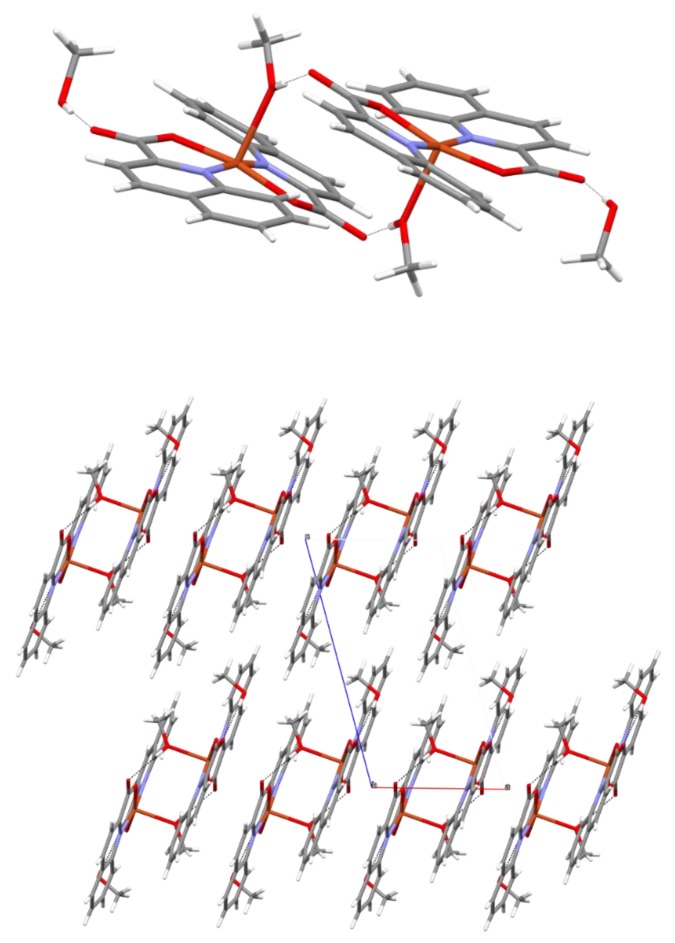
Hydrogen bonds in [Cu(quin)_2_(CH_3_OH)]∙CH_3_OH (**1**). Top drawing: The O–H⋯COO^−^ interactions (dotted lines) link a pair of complex molecules and two solvent molecules of methanol. Lower drawing: The alignment of dimers is quasi-parallel.

Both types of methanol molecules participate in hydrogen bonding interactions (Figure 2). The methanol ligand is hydrogen-bonded to the carboxylate of an adjacent complex molecule. A centrosymmetric dimer, {Cu(quin)_2_(CH_3_OH)}_2_, is thereby formed. To this dimer, two solvent molecules of methanol are attached via O–H⋯COO^−^ hydrogen bonds. The dinuclear assemblies pack in the crystal lattice in an interesting fashion: all the quinaldinates are nearly parallel and are coplanar with the 1 0 −1 lattice plane. *π*⋯*π* stacking interactions may be recognized between the aromatic planes.

**Figure 3:**
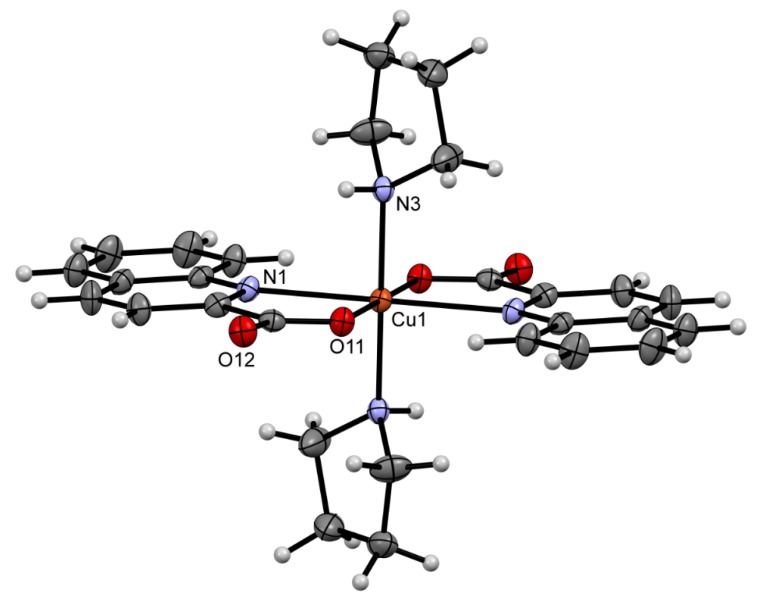
ORTEP drawing of one complex molecule in [Cu(quin)_2_(pyro)_2_] (**2**). Displacement ellipsoids are drawn at the 50% probability level. Hydrogen atoms are shown as spheres of arbitrary radii.

**Figure 4:**
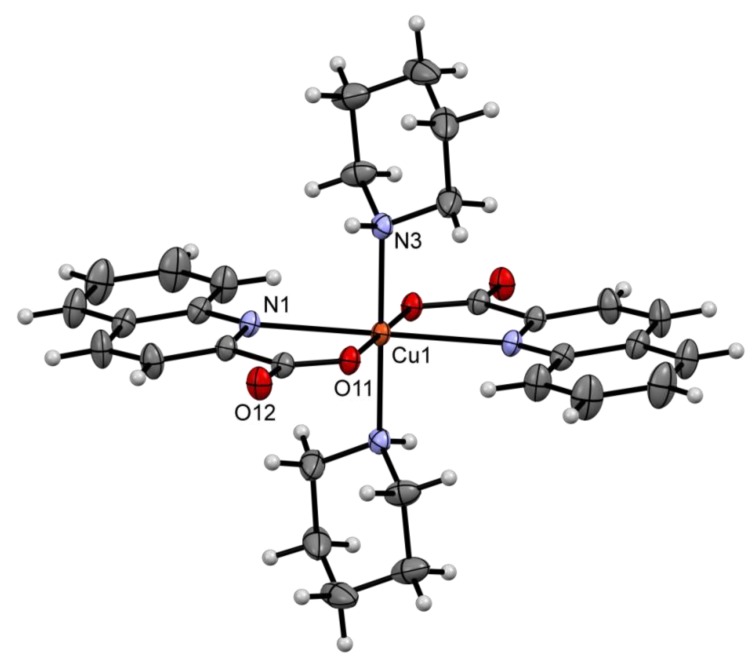
ORTEP drawing of one complex molecule in [Cu(quin)_2_(pipe)_2_] (**7**). Displacement ellipsoids are drawn at the 50% probability level. Hydrogen atoms are shown as spheres of arbitrary radii.

**Figure 5:**
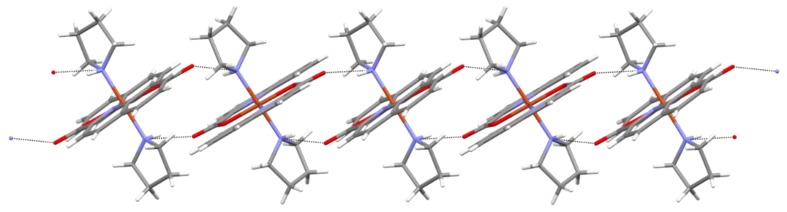
Section of a chain in the structure of [Cu(quin)_2_(pyro)_2_] (**2**).

The structures of [Cu(quin)_2_(pyro)_2_] (**2**), [Cu(quin)_2_(morph)_2_] (**4**) and [Cu(quin)_2_(pipe)_2_] (**7**) are similar: the coordinatively saturated copper(II) centre is six-coordinate with two bidentate chelating quinaldinates and two amine ligands in a relative *trans* disposition (Figure 3 and Figure 4). The N_4_O_2_ donor set occupies vertices of a distorted octahedron. A frequently-encountered “4 + 2” pattern in the coordination bonds may be observed with the longest bonds to the quinaldinate nitrogen atoms. As the bite angle of the chelating ligand is limited, the distortion is restricted. The complex molecules of **2**, **4**, and **7** are centrosymmetric. Interestingly, in the structures of all, the asymmetric unit contains two halves of two complex molecules. For each pair, almost the same metric parameters are displayed with minor differences existing in the orientation of amine ligands. The similarity in the complex molecules of **2**, **4**, and **7** are reflected in their packing arrangements. For all, the complex molecules are hydrogen-bonded via N–H⋯COO^−^ interactions into infinite chains. Within such a chain, each complex molecule forms four hydrogen bonds with two adjacent molecules. A section of a chain of hydrogen-bonded [Cu(quin)_2_(pyro)_2_] molecules in **2** is shown in Figure 5.

The [Cu(quin)_2_(pipe)_2_] complex also crystallizes with solvent molecules of acetonitrile or propionitrile, [Cu(quin)_2_(pipe)_2_]∙CH_3_CN (**8**) and [Cu(quin)_2_(pipe)_2_]∙CH_3_CH_2_CN (**9**), with their structures being isomorphous. The metric parameters of the complex molecules in **8** and **9** are essentially the same and not different from those of **7**. The supramolecular connectivity is similar to that in **7**: the [Cu(quin)_2_(pipe)_2_] molecules are linked in all three structures via N–H⋯COO^−^ hydrogen bonds into chains. A close survey reveals an important difference between the chain structure of **7** and **8** (or **9**). In **7**, the molecules constituting the chains are in two different orientations. Conversely, the molecules in **8** are fully aligned. Dissimilarities in the chains impart different packing motifs. The packing of chains in **7** is dense with moderately short *π*⋯*π* stacking interactions [an arene⋯arene type, *Cg*⋯*Cg* = 3.6889(11) Å, dihedral angle = 0.02(9)°, interplanar distance = 3.4213(8) Å, and offset angle = 22.0°] occurring among adjacent chains [60]. In the structures of **8**/**9**, there are no *π*⋯*π* stacking interactions with centroid-centroid distances below 4.0 Å. Furthermore, their packing is such to produce hydrophobic channels that accommodate solvent molecules of acetonitrile or propionitrile. The channels provide a facile escape route for solvent molecules when the crystals are taken out from the mother liquor.

**Figure 6:**
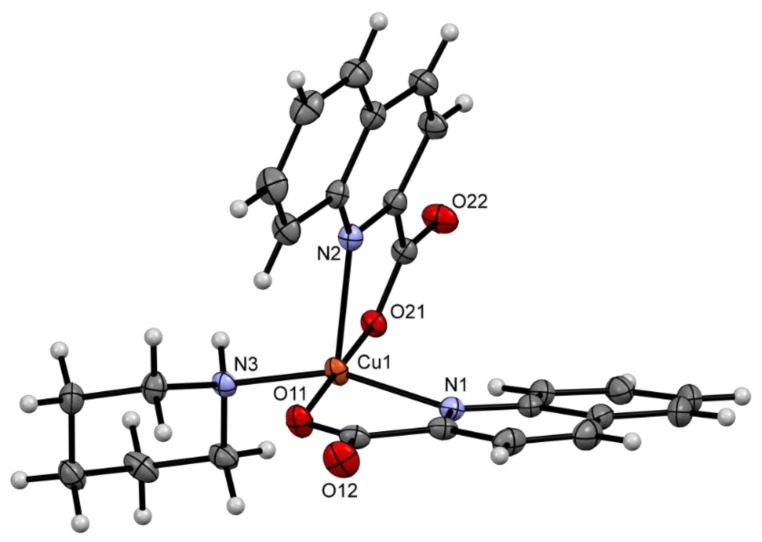
ORTEP drawing of [Cu(quin)_2_(pipe)], a complex molecule of **6**. Displacement ellipsoids are drawn at the 50% probability level. Hydrogen atoms are shown as spheres of arbitrary radii.

We crystallized another copper(II) compound with piperidine, [Cu(quin)_2_(pipe)] (**6**). The [Cu(quin)_2_(pipe)] complex features a five-coordinate metal environment, which consists of two bidentate chelating quinaldinates and a single piperidine ligand (Figure 6). The analysis of the N_3_O_2_ coordination sphere by the method of Addison et al. gave a *τ* descriptor equal to 0.36 [58]. The coordination polyhedron takes the appearance of a distorted square pyramid with *N*,*O*-donors of one quinaldinate, piperidine nitrogen and oxygen of the other quinaldinate in its basal plane, and the remaining quinaldinate nitrogen at its apex. The coordination bonds differ from those determined for a six-coordinate complex, [Cu(quin)_2_(pipe)_2_]. With a smaller number of donors in **6**, the bonds are shorter. The greatest discrepancy is observed in the Cu–N(quin^−^) bonds. In [Cu(quin)_2_(pipe)_2_], the Cu–N bonds exceed 2.4 Å, whereas in a five-coordinate [Cu(quin)_2_(pipe)], these bonds are significantly shorter as they occupy a 2.1065(13) to 2.2635(14) Å interval. The longest one, a result of Jahn–Teller distortion, is formed to the nitrogen at the axial site. With only five coordination bonds in the [Cu(quin)_2_(pipe)] complex, the quinaldinates adopt a more twisted conformation. A non-planarity of the five-membered chelate ring may be given by the Cu–N_ring_–C–C_COO_ torsion angle, which in **6** amounts to 12.94(16)° and 14.82(16)°. The torsion angles in [Cu(quin)_2_(pipe)_2_] are significantly smaller, e.g., the largest one is 7.78(14)° in **9**. For **6**, no short intermolecular interactions may be observed among the [Cu(quin)_2_(pipe)] molecules. Namely, the N–H⋯COO^−^ contact exceeds 3.1 Å. This comes as a surprise as with the amine coordination to copper(II), a partial positive charge of the amine hydrogen is increased and the N–H moiety made a better hydrogen bond donor [61]. In all piperidine complexes, the amine adopts a chair conformation with the NH hydrogen in the axial position.

**Figure 7:**
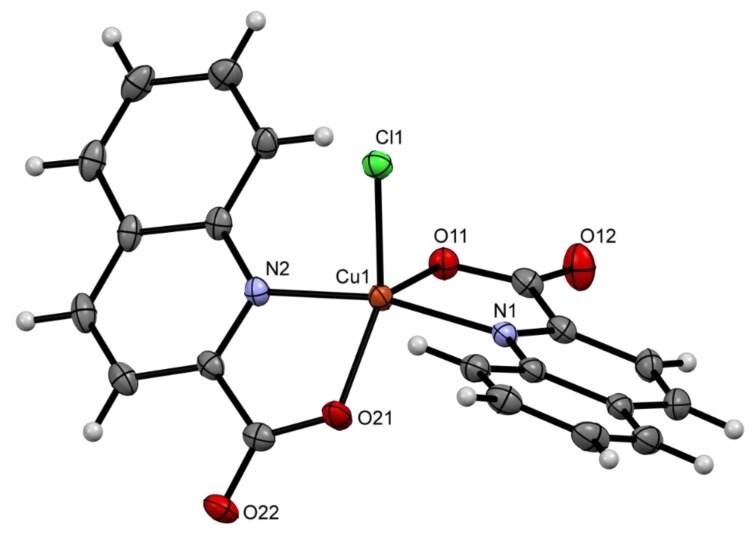
ORTEP drawing of [Cu(quin)_2_Cl]^−^, a complex anion of **3**. Displacement ellipsoids are drawn at the 50% probability level. Hydrogen atoms are shown as spheres of arbitrary radii.

With the structures of (pyroH)[Cu(quin)_2_Cl] (**3**) and (pipeH)[Cu(quin)_2_Cl] (**11**) being isomorphous, a joint description is given. The asymmetric unit comprises of two complex anions, [Cu(quin)_2_Cl]^−^, and two protonated amines as counter-cations. The [Cu(quin)_2_Cl]^−^ ion is a five-coordinate copper(II) with three anionic ligands (Figure 7): two bidentate chelating quinaldinates and a chloride. Metric parameters of the complex anions in the two compounds are essentially the same. The N_2_O_2_Cl donor atoms occupy vertices of a distorted polyhedron that resembles more a trigonal bipyramid than a square pyramid. The values of the *τ* parameter are 0.56 and 0.71 for **3** and 0.51 and 0.77 for **11**, respectively. Dihedral angles between the quinaldinates are 50.65(4)° and 63.68(5)° for **3** and 58.34(2)° and 76.18(3)° for **11**, respectively. With the average for a non-bridging Cu–Cl bond being 2.256 Å [61], the corresponding Cu–Cl bonds in [Cu(quin)_2_Cl]^−^, 2.3332(4)–2.3854(4) Å, are notably longer. Again, the lengthening can be ascribed to the Jahn–Teller effect.

The copper(II) complexes with quinaldinate are very scarce. The literature reports on the structures of only four compounds: [Cu(quin)_2_(H_2_O)] [27] that we used as a starting material, [Cu(quin)X] (X^−^ = Cl^−^ or Br^−^) [24,25] and [Cu(quin)(Hquin)(benzoate)] [62]. A direct comparison of the bonding pattern in our compounds can be made only with the aqua complex. In the case of the other three, either the coordination manner of the ligand is more complex or a different form of quinaldinic acid serves as a ligand. The [Cu(quin)X] compounds feature a quinaldinate ligand bound via all three donor atoms to two metal ions and thereby serving as a bridging ligand, whereas in [Cu(quin)(Hquin)(benzoate)] both the quinaldinate and the quinaldinic acid are coordinated in a *N,O*-bidentate chelating manner and cannot be distinguished because of the symmetry. In [Cu(quin)_2_(H_2_O)], the quinaldinate binds with the Cu–O bonds of 1.954(3) and 1.962(3) Å and slightly longer Cu–N bonds, 2.012(3) and 2.014(3) Å [27]. The same, longer Cu–N than Cu–O bonds, is true for our compounds. In our series, the Cu–O distances do not occupy a wide interval (Table 1). Conversely, the Cu–N bonds vary a lot, from the shortest 1.9865(17) Å, observed in (pyroH)[Cu(quin)_2_Cl] (**3**), to the longest 2.4161(14) Å, observed in [Cu(quin)_2_(pipe)_2_] (**7**). As stated above, the longer one is a result of the Jahn–Teller effect. As exemplified by the [Cu(quin)_2_(pipe)] (**6**) and [Cu(quin)_2_(pipe)_2_] (**7**) pair, the bond lengths are also dependent upon the number of coordination bonds: a five-coordinate complex **6** displays shorter bonds. The influence of the nature of the ligands is demonstrated by [Cu(quin)_2_Cl]^−^, complex anions of **3** and **11**, and [Cu(quin)_2_(CH_3_OH)], a complex molecule of **1**. Although both complexes are five-coordinate copper(II) species, the bonds differ. When all ligands are negatively charged as in [Cu(quin)_2_Cl]^−^, the bonds are shorter.

The amine-to-copper(II) bond lengths in our compounds are rather similar. In a series of piperidine complexes, the one in a five-coordinate [Cu(quin)_2_(pipe)] (**6**) is slightly shorter than the ones in the [Cu(quin)_2_(pipe)_2_] compounds **7**, **8**, and **9**. On the whole, the bonds compare well with those in related compounds. Rare copper(II) complexes with pyrrolidine show the bond length to be dependent upon the location of the donor. In [Cu(Ibu)_2_(pyro)_2_]∙H_2_O (Ibu^−^ = a deprotonated form of ibuprofen) with a square-planar distribution of donors, the Cu–N bond is 1.998(2) Å [63]. The pyrrolidine ligands of [(pyro)_3_Cu(*μ*_2_-OH)_2_Cu(pyro)_3_]^2+^, which occupy equatorial sites in a distorted square-pyramid are at 2.035(1)–2.062(1) Å, whereas the apical one binds at 2.329(1)–2.344(1) Å [64]. Similarly, the Cu–N bond in related copper(II) piperidine complexes can be as short as 2.028(2) Å in case of a square-planar geometry [65], whereas it lengthens to 2.259(4) Å in a square-pyramidal species with amine located at the axial site [66]. Morpholine typically binds in a monodentate manner through nitrogen, as exemplified by the [Cu(diketonate)(morph)_2_X] (X = Cl^−^, SCN^−^ or NO_3_^−^) series with bond lengths in the 2.027(2)–2.075(2) Å range [61]. Rarely, morpholine realizes a bidentate bridging coordination, which involves nitrogen and oxygen donors with the Cu–N bond around 2.02 Å and the Cu–O bond exceeding 2.4 Å [67,68].

**Figure 8:**
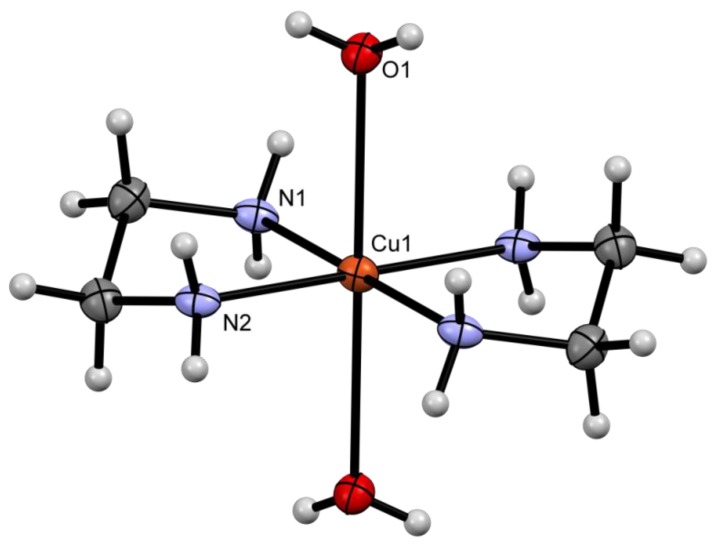
ORTEP drawing of [Cu(en)_2_(H_2_O)_2_]^2+^, a complex cation of **5**. Displacement ellipsoids are drawn at the 50% probability level. Hydrogen atoms are shown as spheres of arbitrary radii.

**Figure 9:**
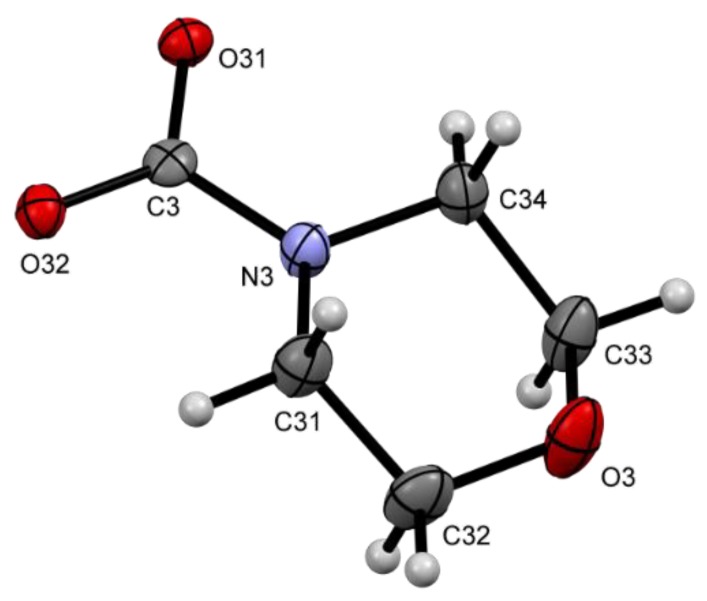
ORTEP drawing of morphCOO^−^, a counter-anion of **5**. Displacement ellipsoids are drawn at the 50% probability level. Hydrogen atoms are shown as spheres of arbitrary radii.

The copper(II) ion in *trans*-[Cu(en)_2_(H_2_O)_2_](morphCOO)_2_ (**5**) features a six-coordinate environment, which comprises of two bidentate chelating ethylenediamine ligands and two water molecules (Figure 8). The distribution of the N_4_O_2_ donors resembles best an elongated octahedron whose square-plane is defined by the ethylenediamine donor atoms and the water oxygen atoms occupying its axial sites. The Cu–N bond lengths are in the 2.0142(15)–2.0200(15) Å range, whereas the Cu–O bond is 2.5384(14) Å. Since the complex is centrosymmetric, one ethylenediamine ligand is in *δ* and the other in a *λ* configuration [5]. The overall metric parameters of the copper(II) complex in **5** do not differ from those in other *trans*-[Cu(en)_2_(H_2_O)_2_]^2+^ compounds [69,70,71,72]. The morphlycarbamate, the counter-anion of **5**, is in a chair conformation (Figure 9). Its nitrogen atom has a partial *sp*^2^ character with the exterior CNC angles being close to 120°. Due to the ring constraints, the interior angle is smaller, 114.37(15)°. The NC_3_ group is not strictly planar as its nitrogen atom lies ca. 0.17 Å out of the carbon atoms plane. Recent survey of the CSD revealed only three compounds with morphylcarbamate ions [38,40,73]. The metric parameters of the morphCOO^−^ ion of **5** are very similar to those.

**Figure 10:**
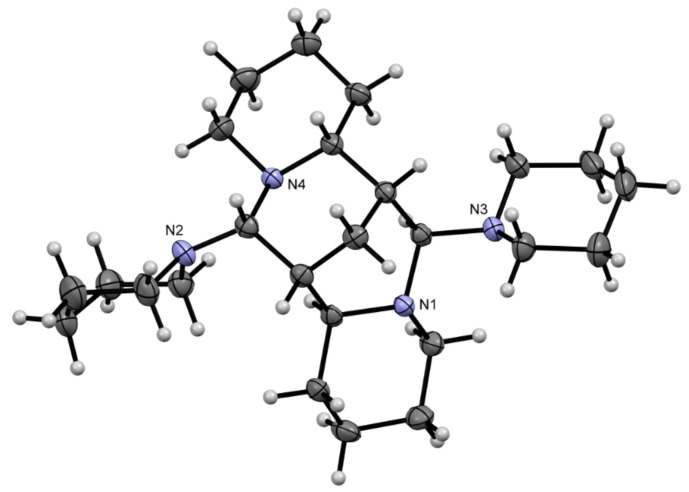
ORTEP drawing of polycyclic piperidine compound **10**. Displacement ellipsoids are drawn at the 50% probability level. Hydrogen atoms are shown as spheres of arbitrary radii.

The cyclic compound **10**, 6,13-di(piperidin-1-yl)dodecahydro-2*H*,6*H*-7,14-methanodipyrido[1,2-*a*:1′,2′-*e*][1,5]diazocine, contains four nitrogen atoms, which are part of six six-numbered rings (Figure 10). Four rings are joined together in a chain-like manner. The remaining two rings are attached via their nitrogen atoms, N2 and N3, to the fused part. The joined rings share two nitrogen atoms among them, N1 and N4. The fused part of the molecule may be viewed as two piperidine rings linked with five carbon atoms. All nitrogen atoms are in trigonal-pyramidal environments, whereas the carbon atoms are *sp*^3^-hybridized and in tetrahedral environments. Six carbon atoms, all belonging to the central two rings, are chiral. Five rings are in the usual chair conformation, whereas one, the internal N1 ring, is in the boat conformation. The dimensions of the heteronuclear rings were compared to dimensions of piperidine in [Cu(quin)_2_(pipe)] (**6**). The N2 and N3 rings display somewhat shorter C–N bonds, i.e., 1.447(2)–1.463(2) Å vs. 1.488(2)–1.489(2) Å observed for **6**. The same observation pertains to the peripheral N1 and N4 rings. Significant lengthening was observed for the C–C bonds in the inner two rings, i.e., the longest bond amounts to 1.559(2) Å for **10** vs. 1.526(3) Å for **6**. In the solid state structure of **10**, the molecules are held together by weak intermolecular interactions.

### 2.3. Infrared Spectra

The infrared spectra of title compounds are dominated by the absorptions of the quinaldinate ligands. Both the positions and intensities of the bands that originate from the normal modes of quinaldinates are very similar. Without exceptions, all spectra show a set of four absorption peaks with a medium to strong intensity at 1568, 1513, 1461, and 1435 cm^−1^, as exemplified by the spectrum of [Cu(quin)_2_(CH_3_OH)]∙CH_3_OH (**1**). By far, the most intense bands pertain to the *ν*_as_(COO^−^) and *ν*_s_(COO^−^) absorptions. Their positions in the spectra of our compounds are listed in Table 2. These are in good agreement with the assignments reported previously for related quinaldinate complexes of other transition metals, as exemplified by 1642 and 1366 cm^−1^ found for [Zn(quin)_2_(1-methylimidazole)_2_] [74]. The *ν*_as_(COO^−^) absorption occupies, in view of a great similarity of compounds, a surprisingly wide range, from 1676 cm^−1^ observed for [Cu(quin)_2_(pipe)] (**6**) to 1611 cm^−1^ for (pipeH)[Cu(quin)_2_Cl] (**11**). The spectra of **7**, **8**, and **9**, compounds that contain the [Cu(quin)_2_(pipe)_2_] complex, feature the *ν*_as_(COO^−^) absorption at 1624–1626 cm^−1^. Although the *ν*_as_(COO^−^) frequency appears to be sensitive to the immediate environment of the Cu^2+^ ion, a more direct correlation exists with the involvement in hydrogen bonds. The highest frequency is observed for [Cu(quin)_2_(pipe)] (**6**), the only compound in the series with carboxylate moiety not engaged in strong intermolecular interactions. Large splitting values *∆*, i.e., a difference between the *ν*_as_(COO^−^) and *ν*_s_(COO^−^) frequencies, in the 255–335 cm^−1^ range in the spectra of our compounds are as expected for a monodentate carboxylate coordination [75].

The presence of the amine ligands is confirmed by an absorption band of medium intensity at ca. 3200 cm^−1^ whose origin lies in the *ν*(N–H) vibration. In addition, several weaker bands in the 2990–2850 cm^−1^ range, due to the stretching vibrations of the aliphatic C–H bonds, may be seen. The position of the *ν*(N–H) band in piperidine compounds **6**–**9** shows correlation to the lengths of intermolecular contacts that involve the NH group. [Cu(quin)_2_(pipe)] (**6**) reveals a band at 3232 cm^−1^, at higher energy when compared to ca. 3206 cm^−1^ (observed for **8** and **9**) or 3170 cm^−1^ (**7**). The NH moiety in **6** does not participate in stronger intermolecular interactions. The opposite is true for **7**, **8**, and **9** with NH engaged in a hydrogen bonding interaction with the carboxylate oxygen.

The spectra of the amine salts are markedly different from those of the parent amine ligands. In the spectra of pyrrolidinium and piperidinium salts of the [Cu(quin)_2_Cl]^−^ ion, the region of both *ν*(N–H) and *ν*(C–H) absorptions is masked by two broad bands centred at ca. 2950 and 2460 cm^−1^. Their intensity and shape reflect an extensive hydrogen bonding that involves the NH_2_^+^ group. In addition, the spectra of both **3** and **11** reveal a peak that protrudes from the *ν*_as_(COO^−^) band at ca. 1590 cm^−1^. The latter appears in the region for the NH_2_^+^ bending vibrations [76].

The infrared spectrum of [Cu(quin)_2_(pipe)_2_]∙CH_3_CH_2_CN (**9**) revealed a weak band at 2244 cm^−1^, which can be attributed to the *ν*(C≡N) of the lattice propionitrile. The absence of the absorption peak at this wavenumber in the spectrum of [Cu(quin)_2_(pipe)_2_]∙CH_3_CN (**8**) is consistent with a rapid loss of acetonitrile on removing the crystalline solid from the mother liquor.

### 2.4. Conversion of [Cu(quin)_2_(CH_3_OH)]∙CH_3_OH *(**1**)* into the Aqua Complex

**Figure 11:**
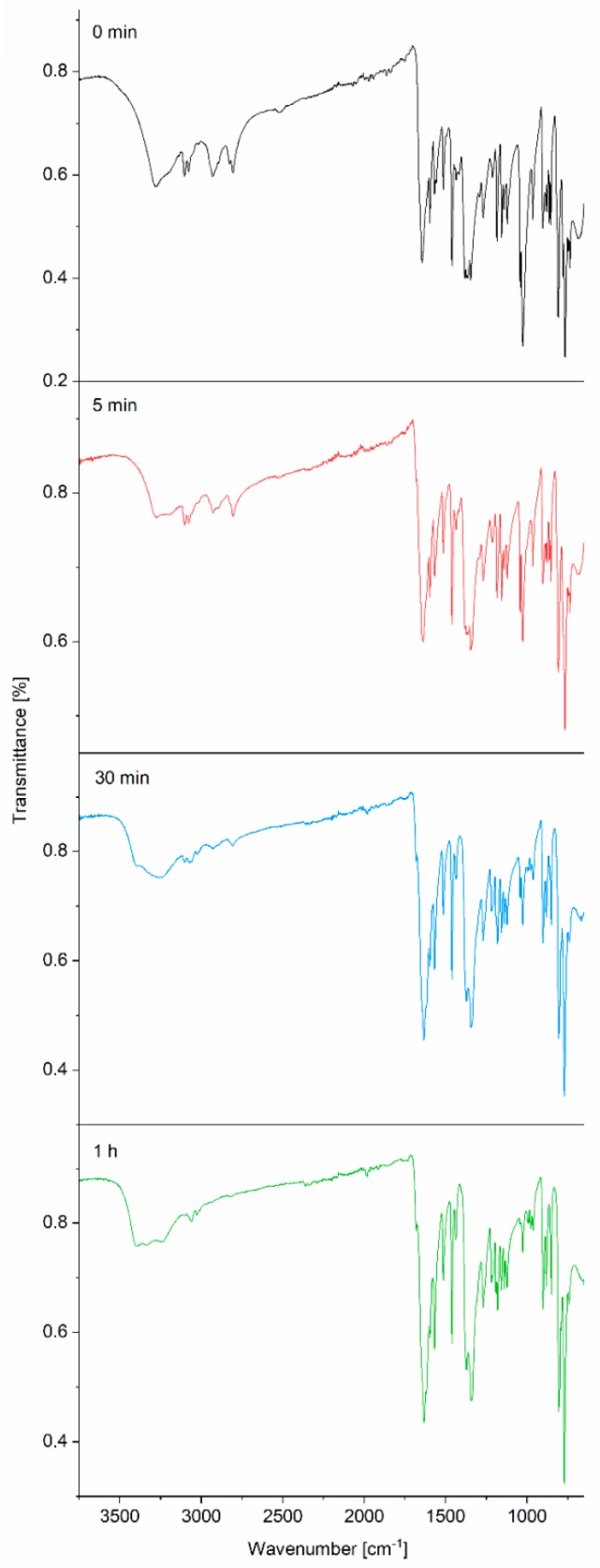
A time evolution of the IR spectra of [Cu(quin)_2_(CH_3_OH)]∙CH_3_OH (**1**). Color code: black—0, red—5, blue—30 and green—60 min.

The conversion was monitored by IR spectroscopy (Figure 11). The infrared spectrum of [Cu(quin)_2_(CH_3_OH)]∙CH_3_OH (**1**) features absorption bands at 3276, 2929, 2828, 2807, 1042, and 1026 cm^−1^ whose origin lies in the vibrations of methanol [76]. Their intensity rapidly diminished with time, confirming the loss of weakly-bound methanol. A 5-min exposure of the sample to the air atmosphere resulted in a spectrum with the intensity of methanol peaks reduced by ca. 30%. With further exposure, the methanol peaks completely disappeared. Instead, a broad band at ca. 3300 cm^−1^, ascribed to the *ν*(O–H) vibrations of water, started to gain in intensity. In addition, a shift of the *ν*_as_(COO^−^) band from 1645 cm^−1^ for [Cu(quin)_2_(CH_3_OH)]∙CH_3_OH (**1**) to 1631 cm^−1^ for the product, [Cu(quin)_2_(H_2_O)], could be observed. Different *ν*_as_(COO^−^) frequencies for the methanol and aqua complexes are yet another demonstration of the influence of the hydrogen bonding over the position of the *ν*_as_(COO^−^) bands, described in the preceding section. In both compounds, the neutral *O*-donor is engaged in intermolecular interactions with the carboxylate moiety. In the aqua complex [27], the corresponding O⋯O contacts are by ca. 0.1 Å shorter than in the methanol complex. With stronger hydrogen bonds in [Cu(quin)_2_(H_2_O)], the *ν*_as_(COO^−^) frequency is shifted to a lower energy. The conversion into the aqua complex was completed after one hour. An explanation for the facile conversion was sought for in the solid state structures of the methanol and aqua complexes. Although no apparent reasons could be disclosed, certain structural features were brought to our attention. In spite of the likeness of the *O*-ligands, the overall structures are markedly different. The complexes have a different spatial distribution of the ligands in the first coordination sphere of the metal ion. The quinaldinates of [Cu(quin)_2_(CH_3_OH)]∙CH_3_OH (**1**) are nearly parallel, whereas those in [Cu(quin)_2_(H_2_O)] are at an angle of approximately 58° [27]. Different overall shapes of complex molecules impart different packing arrangements. Whereas the solid state structure of **1** consists of dimeric {[Cu(quin)_2_(CH_3_OH)]_2_(CH_3_OH)_2_} assemblies with their quinaldinates parallelly aligned and held together by weak *π*⋯*π* stacking interactions, the [Cu(quin)_2_(H_2_O)] molecules are linked with stronger intermolecular contacts, i.e., the O–H⋯COO^−^ hydrogen bonds, into an infinite 2D-array. The main stimulus for the conversion probably lies in the fact that water fulfils the role of a stronger ligand and of a better hydrogen bond donor than methanol. The end result is the aqua complex with a very stable structure. Weak intermolecular forces and the apparent ease of the spatial rearrangement of quinaldinate ligands in **1** must also be recognized as the contributing factors.

## 3. Materials and Methods

### 3.1. General

All manipulations and procedures were conducted in air. First reactions were carried out with an old batch of piperidine and morpholine in originally sealed bottles, sold 30 years ago by Ventron. During the course of this study, new chemicals were purchased from Sigma Aldrich. With the exception of acetonitrile, the chemicals were used as received. Acetonitrile was dried over molecular sieves, following the published procedure [77]. The IR spectra were recorded from 4000 to 400 cm^−1^ with a Bruker Alpha II FT-IR instrument. The solid samples were analyzed on the single reflection ATR accessory. Elemental analyses (C, H, N) were performed by the in-house facility on a Perkin-Elmer 2400 II instrument. The thermal analysis of [Cu(quin)_2_(CH_3_OH)]∙CH_3_OH (**1**) was performed on a Mettler Toledo TG/DSC 1 instrument. Crystals of **1** were removed from the mother liquor, placed for a few seconds on a filter paper and then into a platinum crucible. Their mass was 4.1823 mg. The carrier gas was argon at a flow rate of 50 mL min^−1^. The sample was heated from 20 to 800 °C at a rate of 10 °C min^−1^. The baseline was subtracted. PXRD data for [Cu(quin)_2_(H_2_O)], our starting material, were collected on a PANalytical X’Pert PRO MD diffractometer using a Cu-K*_α_* radiation (*λ* = 1.5406 Å).

### 3.2. Synthetic Procedures

#### 3.2.1. Synthesis of the Copper(II) Starting Material

A teflon container was loaded with copper(II) acetate hydrate (100 mg, 0.50 mmol of copper) and quinaldinic acid (173 mg, 1.00 mmol). Methanol (15 mL) was added. The container was closed and inserted into a steel autoclave. The autoclave was heated for 24 h at 105 °C. The reaction vessel was then allowed to cool slowly to room temperature. Large turquoise crystals of [Cu(quin)_2_(CH_3_OH)]∙CH_3_OH (**1**) were collected by filtration. Mass of the dried product was 185 mg. Yield: 0.43 mmol, 86%. The crystals of [Cu(quin)_2_(CH_3_OH)]∙CH_3_OH (**1**) turned opaque almost instantaneously when removed from the mother liquor. The infrared spectrum of the opaque crystals was identical with the spectrum of the known aqua complex, i.e., [Cu(quin)_2_(H_2_O)] [27], whose identity was confirmed by checking unit cell dimensions on the X-ray diffractometer. PXRD of the aged compound was a superposition of the calculated pattern for the known [Cu(quin)_2_(H_2_O)] and several peaks, which belonged to an unidentified crystalline phase. By chance, we obtained single crystals of the other polymorph of [Cu(quin)_2_(H_2_O)] with the following unit cell parameters: monoclinic *C* 2, *a* = 12.6260(3), *b* = 9.5418(3), *c* = 14.8143(5) Å, *α* = 90, *β* = 97.037(2), *γ* = 90° and *V* = 1771.31(9) Å^3^. Owing to the low-quality of the X-ray data, the *R*_1_ and *wR*_2_ residuals remained large. Nevertheless, its composition, [Cu(quin)_2_(H_2_O)], was not questionable. The offending peaks in the measured PXRD of the aged compound were found to belong to the second polymorph (please see Figure S1). The calculated yield and the elemental analysis data referred to the aqua complex. Found C, 56.40; H, 3.30; N, 6.56%. C_20_H_14_CuN_2_O_5_ (425.88 g mol^−1^) required C, 56.40; H, 3.31; N, 6.58%. IR of [Cu(quin)_2_(CH_3_OH)]∙CH_3_OH (**1**) (ATR, cm^−1^): 3276m (broad), 3104m, 3078m, 2929m, 2828w, 2807m, 1645vvs, 1597vs, 1568s, 1558s, 1513s, 1461vs, 1435w, 1418w, 1380vs, 1371vs, 1364vs, 1346vs, 1292w, 1269m, 1212w, 1184s, 1155s, 1142m, 1120s, 1042vs, 1026vvs, 965m, 903s, 885s, 876s, 863s, 853s, 809vs, 779s, 766vvs, 747m, 737s, 685m (broad), 642s, 627s, 611vs, 577w, 522m, 500s, 483m, 433vs. IR of [Cu(quin)_2_(H_2_O)] (ATR, cm^−1^): 3260m (broad), 3060w, 1631vvs, 1616vvs, 1597s, 1568s, 1513m, 1461s, 1436m, 1372vvs, 1344vvs, 1269m, 1219m,1182m, 1156w, 1139w, 1121w, 1042w, 1026m, 977w, 962w, 902s, 882m, 850m, 806vs, 770vvs, 744w, 672m, 644m, 605s, 570m, 523m, 496s, 432w, 403s. Thermal analysis of [Cu(quin)_2_(CH_3_OH)]∙CH_3_OH (**1**): The compound decomposed in two endothermic stages over the temperature range 20 to 800 °C. It started to lose mass almost immediately upon heating. By 115 °C, the mass stabilized. In the first decomposition step, the loss amounted to 13.27% of the initial mass. The experimental value agreed with the calculated one for the release of two methanol molecules per formula unit, 13.58%. The residue {Cu(quin)_2_} displayed a region of stability up to 260 °C when the second, major degradation process set in. The 260–520 °C temperature interval witnessed a 61.95% reduction of mass. On further heating to 800 °C, a continuous decrease in the mass of the solid residue may be observed. The TG and DSC curves are given in Figure S2.

#### 3.2.2. Synthesis of [Cu(quin)_2_(pyro)_2_] (**2**)

Pyrrolidine (1 mL) was added to acetonitrile (10 mL) in an Erlenmeyer flask. To this mixture, [Cu(quin)_2_(H_2_O)] (100 mg, 0.23 mmol) was added. The flask was closed and left stirring at ambient conditions for 3 days. Precipitate of a light blue color was filtered off and washed with the hexanes. The filtrate of a very light blue color was kept in a closed container in the refrigerator. Within a week, tiny, blue block-shaped crystals of **2** grew from the solution. Yield (first isolated solid): 92 mg, 0.17 mmol, 71%. Found C, 60.95; H, 5.22; N, 10.05%. C_28_H_30_CuN_4_O_4_ (550.11 g mol^−1^) requires C, 61.13; H, 5.50; N, 10.18%. IR (ATR, cm^−1^): 3170m, 3054w, 3044w, 2983w, 2971w, 2946w, 2868w, 1620vvs, 1565s, 1507m, 1465m, 1450w, 1433w, 1363vs, 1345m, 1321w, 1275w, 1262w, 1219m, 1172vs, 1157s, 1122s, 1069m, 1057w, 1033w, 1020m, 988w, 974w, 958m, 946w, 920vs, 894vs, 873m, 849vs, 799vvs, 778vvs, 773vvs, 743m, 734m, 633s, 628s, 600s, 551w, 523m, 493s, 476w.

#### 3.2.3. Synthesis of (pyroH)[Cu(quin)_2_Cl] (**3**)

A small amount of [Cu(quin)_2_(pyro)_2_] (**2**) (ca. 10 mg) was dissolved in dichloromethane (2 mL). The resulting blue colored solution in a small glass tube was carefully layered with diethyl ether. The tube was stoppered and left to stand at ambient conditions. Within a week, the solution acquired a yellow-green color and a small amount of yellow-green crystals of **3** deposited on the glass walls. **3** was available in very small quantities, sufficient for the X-ray analysis on a single crystal and IR spectroscopy. IR (ATR, cm^−1^): ca. 3000m (broad), 2979m, 2459m (broad), 1622vs, 1615vs, 1595s, 1566vs, 1512m, 1460s, 1435m, 1374vs, 1365s, 1357s, 1344vs, 1261m, 1219w, 1208w, 1178m, 1153m, 1120w, 1114m, 1034w, 1024w, 983w, 972w, 957w, 896m, 884m, 875m, 862w, 849m, 799vs, 774vs, 735m, 647s, 630m, 604s, 567m, 523m, 499s, 404s.

#### 3.2.4. Synthesis of [Cu(quin)_2_(morph)_2_] (**4**)

Morpholine (1.5 mL) was added to acetonitrile (10 mL) in an Erlenmeyer flask. To this mixture, [Cu(quin)_2_(H_2_O)] (100 mg, 0.23 mmol) was added. The flask was closed and left stirring at ambient conditions for 24 h. Precipitate of a light blue color was filtered off and washed with the hexanes. Yield: 89 mg, 0.15 mmol, 65%. Found C, 57.70; H, 5.28; N, 9.61%. C_28_H_30_CuN_4_O_6_ (582.11 g mol^−1^) requires C, 57.77; H, 5.19; N, 9.62%. IR (ATR, cm^−1^): 3162m, 2975w, 2962w, 2950m, 2935w, 2857w, 1618vvs, 1566s, 1551m, 1506m, 1468m, 1460m, 1439s, 1377vs, 1363vvs, 1331w, 1323w, 1306w, 1259m, 1218m, 1205w, 1199w, 1175m, 1170m, 1158m, 1115s, 1097vs, 1070m, 1062m, 1034s, 1017w, 956w, 895m, 881vvs, 852s, 800vvs, 776vvs, 748w, 740m, 649m, 632s, 627s, 601s, 551w, 523w, 498s, 490m, 479w, 438w.

#### 3.2.5. Preparation of Single Crystals of [Cu(quin)_2_(morph)_2_] (**4**) and *trans*-[Cu(en)_2_(H_2_O)_2_](morphCOO)_2_ (**5**)

A modified procedure for the synthesis of **4** was used. Morpholine (1.5 mL) was added to the mixture of acetonitrile (7 mL) and methanol (2 mL) in an Erlenmeyer flask. To this mixture, [Cu(quin)_2_(H_2_O)] (100 mg, 0.23 mmol) was added. The flask was closed and left stirring at ambient conditions for 24 h. Light blue precipitate, compound **4**, was filtered off and washed with the hexanes. Mass of the dried solid was 85 mg. A pale blue filtrate was allowed to stand in the air with the evaporative loss of solvent for 24 h. Afterwards, the flask was closed and placed in the refrigerator. Within a week, two crystalline phases, blue, needle-shaped crystals of **4** and violet, very thin needle-shaped crystals of **5**, grew from the solution. Both compounds were identified by single crystal X-ray diffraction studies.

#### 3.2.6. Synthesis of [Cu(quin)_2_(pipe)] (**6**)

Copper(II) chloride dihydrate (80 mg, 0.47 mmol) was added to the solution of piperidine (1.5 mL) in acetonitrile (10 mL) in an Erlenmeyer flask. To this mixture, quinaldinic acid (173 mg, 1.00 mmol) was added. The flask was closed and stirred at ambient conditions until all solids were consumed. The stirring did not last longer than 5 to 10 min. The resulting solution of a green color was placed into the refrigerator. Large crystals of a royal blue color grew overnight. The crystalline product was filtered off and washed with the hexanes. Yield: 109 mg, 0.22 mmol, 47%. Found C, 60.71; H, 4.78; N, 8.32%. C_25_H_23_CuN_3_O_4_ (493.01 g mol^−1^) requires C, 60.90; H, 4.70; N, 8.52%. IR (ATR, cm^−1^): 3232m, 3101w, 3060w, 3050w, 3016w, 2948w, 2928m, 2861w, 1676m, 1651vvs, 1593m, 1565m, 1510m, 1458s, 1434m, 1355vvs, 1341vvs, 1296w, 1272w, 1260w, 1253w, 1219w, 1192m, 1175s, 1150m, 1143m, 1111m, 1080s, 1044m, 1025m, 1005s, 978m, 961m, 949m, 896s, 874s, 866s, 853s, 813s, 800vvs, 788vvs, 778vvs, 755s, 738s, 635s, 607s, 524s, 504m, 495m, 482m, 456w, 419vs.

#### 3.2.7. Reaction of **6** with Piperidine

[Cu(quin)_2_(pipe)] (**6**) (50 mg, 0.10 mmol) was added to the solution of piperidine (0.5 mL) in acetonitrile (10 mL). The container was closed and its contents left stirring at ambient conditions for two days. All the solid was consumed meanwhile and the solution acquired a green color. The solution was placed in the refrigerator. Large, blue-colored crystals of [Cu(quin)_2_(pipe)_2_] (**7**) grew overnight. Their composition was confirmed by their IR spectrum and determination of unit cell parameters on the X-ray diffractometer.

#### 3.2.8. Synthesis of [Cu(quin)_2_(pipe)_2_] (**7**)

A teflon container was loaded with [Cu(quin)_2_(H_2_O)] (50 mg, 0.12 mmol), acetonitrile (5 mL), and piperidine (1.5 mL). The container was closed and inserted into a steel autoclave. The autoclave was heated for 24 h at 105 °C. The reaction vessel was then allowed to cool slowly to room temperature. Yellow-green solution was stored in a closed container in the refrigerator. Within 1 week, large blue block-like crystals of **7** were obtained. The crystals were isolated by filtration. Yield: 41 mg, 0.07 mmol, 61%. Found C, 62.05; H, 6.00; N, 9.64%. C_30_H_34_CuN_4_O_4_ (578.15 g mol^−1^) requires C, 62.32; H, 5.93; N, 9.69%. IR (ATR, cm^−1^): 3170m, 3043w, 3014w, 2964w, 2933m, 2852m, 1626vvs, 1566s, 1506m, 1463s, 1444s, 1364vvs, 1358vvs, 1346vs, 1276m, 1259w, 1215m, 1202m, 1191m, 1172vs, 1157s, 1150m, 1111m, 1092s, 1050m, 1044m, 1023vs, 1010vs, 957m, 944m, 893vs, 877vvs, 851vvs, 814m, 801vvs, 771vvs, 745w, 734m, 631s, 626m, 615m, 601s, 551w, 522m, 497s, 473m.

#### 3.2.9. Reaction of [Cu(quin)_2_(H_2_O)] with Piperidine at Ambient Conditions

Piperidine (1.5 mL) was added to acetonitrile (10 mL) in an Erlenmeyer flask. To this mixture, [Cu(quin)_2_(H_2_O)] (100 mg, 0.23 mmol) was added. The flask was closed and left stirring at ambient conditions for 4 days. The color turned from green to deep red-brown and all the solid was consumed. The solution was placed in the refrigerator. Within an hour or after a couple of days, a copious amount of green crystalline material deposited. The solid consisted of at least 2 phases, [Cu(quin)_2_(pipe)_2_] (**7**) and [Cu(quin)_2_(pipe)_2_]∙CH_3_CN (**8**). Their identity was unambiguously confirmed by checking unit cell dimensions on the X-ray diffractometer (in case of **7**) or the complete X-ray diffraction analysis (in case of **8**). The average amount of the dried solid was 85 mg. On exposing a red-brown solution to the air, its color changed instantaneously to green. Within a week, a characteristic odor of the ammonia gas could be detected in the reaction mixtures. In one instance only, the reaction mixture that was kept at 5 °C for 2 months produced apart from [Cu(quin)_2_(pipe)_2_] (**7**) and [Cu(quin)_2_(pipe)_2_]∙CH_3_CN (**8**), a substantial amount of colorless crystals of **10**. The true composition of **10** was identified by X-ray structure analysis. No other analyses could be carried out because of the contamination with copper(II) compounds. Numerous attempts to reproduce the preparation of **10** were not met with success. IR of [Cu(quin)_2_(pipe)_2_]∙CH_3_CN (**8**) (ATR, cm^−1^): 3208m, 3045w, 2940m, 2910m, 2871w, 2850w, 1652w, 1626vvs, 1595w, 1564s, 1505m, 1468m, 1460s, 1454m, 1434w, 1427w, 1355vvs, 1344vvs, 1317w, 1278w, 1253m, 1214w, 1191m, 1169s, 1149m, 1119m, 1086vs, 1048s, 1025s, 1006s, 979w, 956w, 944m, 893s, 876vvs, 848s, 800vvs, 781vvs, 771vvs, 745m, 736m, 630vs, 610m, 598s, 555m, 521s, 501vs, 481w, 469w, 456w, 438w.

#### 3.2.10. Synthesis of [Cu(quin)_2_(pipe)_2_]∙CH_3_CH_2_CN (**9**)

Piperidine (1.5 mL) was added to propionitrile (10 mL) in an Erlenmeyer flask. To this mixture, [Cu(quin)_2_(H_2_O)] (100 mg, 0.23 mmol) was added. The flask was closed and left stirring at ambient conditions for 4 days. The solid was consumed meanwhile and the reaction mixture acquired a dark brown red color. The reaction mixture was placed in the refrigerator. Overnight, the color of the reaction mixture changed to green and a copious amount of blue crystalline material deposited from the solution. The solid was filtered off and washed with the hexanes. *Note.* The crystals of **9** were found to turn opaque when removed from the mother liquor. Yield (dried product): 58 mg, 0.10 mmol, 43%. The calculated yield and the elemental analysis data refer to [Cu(quin)_2_(pipe)_2_]. Found C, 62.46; H, 5.79; N, 9.57%. C_30_H_34_CuN_4_O_4_ (578.15 g mol^−1^) requires C, 62.32; H, 5.93; N, 9.69%.IR (ATR, cm^−1^): 3204s, 3037w, 2985w, 2961w, 2932s, 2871m, 2860m, 2244w, 1657w, 1624vvs, 1591s, 1564vs, 1548m, 1506m, 1460vs, 1451vs, 1438w, 1422w, 1362vvs, 1345vs, 1316m, 1299w, 1274m, 1257m, 1219m, 1193m, 1172vs, 1155m, 1121w, 1111w, 1086vs, 1045m, 1025s, 1010vs, 980w, 958w, 945m, 894s, 876vs, 848vs, 803vvs, 784vvs, 745m, 631vs, 612m, 598vs, 551w, 523m, 501vs, 482w, 472w.

#### 3.2.11. Synthesis of (pipeH)[Cu(quin)_2_Cl] (**11**)

Piperidine (1.5 mL) was added to the mixture of acetonitrile (5 mL) and dichloromethane (5 mL) in an Erlenmeyer flask. To this mixture, [Cu(quin)_2_(H_2_O)] (100 mg, 0.23 mmol) was added. The flask was closed and left stirring at ambient conditions. The solid material immediately dissolved rendering a dark green solution. After 4 days of stirring, the color changed to orange brown and a solid material precipitated. The solids, a mixture of green microcrystalline material, presumably a [Cu(quin)_2_(pipe)_2_] compound, and of colorless crystals of piperidinium chloride, were filtered off. A glass vial containing diethyl ether (5 mL) was carefully inserted into the flask with the orange brown filtrate. The flask was tightly stoppered and stored at ambient conditions. After 2 months, a small amount of yellow green crystals of **11** was manually separated from colorless needle-like crystals of piperidinium chloride. Found C, 56.48; H, 4.68; N, 7.89%. C_25_H_24_ClCuN_3_O_4_ (529.46 g mol^−1^) requires C, 56.71; H, 4.57; N, 7.94%. IR (ATR, cm^−1^): 2948m, 2928m, 2839m, 2803m, 2762m, 2734m, 2526m, 2427w, 1611vvs, 1593vvs, 1564vvs, 1510m, 1459vvs, 1435m, 1367vvs, 1355vvs, 1345vvs, 1314w, 1275w, 1266w, 1219w, 1206w, 1181m, 1154m, 1115m, 1078w, 1059w, 1032m, 1022w, 972w, 954m, 949m, 896s, 883m, 872m, 862m, 850m, 796vvs, 774vvs, 739m, 733m, 646m, 631m, 604vs, 556s, 523m, 500s, 490w, 439s.

### 3.3. X-ray Structure Determination

Single crystal X-ray diffraction data were collected on an Agilent SuperNova diffractometer with molybdenum (Mo-K*_α_*, *λ* = 0.71073 Å) or copper (Cu-K*_α_*, *λ* = 1.54184 Å) micro-focus sealed X-ray source at 150 K. Each crystal was placed on a tip of a glass fiber using silicone grease and then mounted on the goniometer head. Data processing was performed with CrysAlis PRO [78]. The structures were solved with Olex software [79] using ShelXT [80] and refined using the least squares methods in ShelXL [81]. Anisotropic displacement parameters were determined for all non-hydrogen atoms. Details on the second polymorph of [Cu(quin)_2_(H_2_O)] were given in the preceding section. The solvent molecules in [Cu(quin)_2_(pipe)_2_]∙CH_3_CN (**8**) and [Cu(quin)_2_(pipe)_2_]∙CH_3_CH_2_CN (**9**) were disordered over the inversion center. For **9**, the disorder was successfully resolved using PART −1 instruction. In case of **8**, the disorder could not be modelled and the contribution of the disordered solvent to the scattering factors was, therefore, accounted for by the SQUEEZE program [82]. In all structures, the NH or NH_2_^+^ hydrogen atoms and the OH hydrogen atoms of methanol and water were located from difference Fourier maps and refined with isotropic displacement parameters. The remaining hydrogen atoms were added in calculated positions. Programs Platon [83], Ortep [84], and Mercury [85] were used for crystal structure analysis and preparation of figures. Crystallographic data are collected in Table 3 and Table 4. All crystal structures were deposited to the CSD and have been assigned deposition numbers CCDC-1984542 (**1**), -1984543 (**2**), -1984544 (**3**), -1984545 (**4**), -1984546 (**5**), -1984547 (**6**), -1984548 (**7**), -1984549 (**8**), -1984550 (**9**), -1984551 (**10**) and -1984552 (**11**). These data can be obtained free of charge via http://www.ccdc.cam.ac.uk/conts/retrieving.html (or from the CCDC, 12 Union Road, Cambridge CB2 1EZ, UK; Fax: +44 1223 336033; E-mail: deposit@ccdc.cam.ac.uk).

## 4. Conclusions

Reactions of copper(II) quinaldinate under mild conditions with selected alicyclic secondary amines, pyrrolidine, morpholine, and piperidine, produced desired amine complexes with the [Cu(quin)_2_(amine)] or *trans*-[Cu(quin)_2_(amine)_2_] compositions. The {Cu(quin)_2_} structural fragment underwent substitution reactions with ethylenediamine, an impurity in morpholine, producing *trans*-[Cu(en)_2_(H_2_O)_2_](morphCOO)_2_ with morphylcarbamate as counter-anions. The morphCOO^−^ ions and the anionic complex of (pyroH)[Cu(quin)_2_Cl] and (pipeH)[Cu(quin)_2_Cl] give evidence of the amine reactivity towards carbon dioxide or dichloromethane, respectively. Both interfering reactions are known. Despite the high similarity of the used amines, the behavior of piperidine towards copper(II) differed. In acetonitrile, the metal ions were reduced. The active role of the amine in electron transfer was confirmed by the X-ray structure analysis of a polycyclic piperidine derivative, not known prior to this work. Its formation, which probably involves a series of radical reactions, invites further investigation. Such studies are underway.

## Figures and Tables

**Table 1:** Relevant bond lengths [Å] in **1**–**11**.

Compound	Donor Set	*τ ^a^*	Cu–N(quin^−^)	Cu–O(quin^−^)	L	Cu–L
**1**	N_2_O_3_	0.05	2.0994(15), 2.0836(15)	1.9112(12), 1.9081(12)	CH_3_OH	2.2806(14)
**2**	N_4_O_2_	–	2.3648(19), 2.3755(18)	2.0440(15), 2.0279(14)	pyro	2.0312(19), 2.0238(19)
**3**	N_2_O_2_Cl	0.56, 0.71	1.9865(17), 1.9974(17), 1.9896(17), 2.0093(17)	2.0774(15), 1.9947(15), 2.0365(14), 2.0162(14)	Cl^−^	2.3811(6), 2.3483(6)
**4**	N_4_O_2_	–	2.387(3), 2.406(3)	2.010(2), 1.987(2)	morph	2.064(3), 2.074(3)
**6**	N_3_O_2_	0.36	2.1065(13), 2.2635(14)	1.9246(12), 1.9373(11)	pipe	2.0291(13)
**7**	N_4_O_2_	–	2.4161(14), 2.3659(14)	1.9864(12), 2.0035(12)	pipe	2.0727(15), 2.0833(15)
**8**	N_4_O_2_	–	2.3894(13)	1.9839(10)	pipe	2.0899(13)
**9**	N_4_O_2_	–	2.3805(12)	1.9787(10)	pipe	2.0921(12)
**11**	N_2_O_2_Cl	0.51, 0.77	1.9960(12), 2.0262(12), 2.0022(12), 1.9898(13)	2.0327(11), 2.0307(11), 2.0541(11), 2.0046(11)	Cl^−^	2.3332(4), 2.3854(4)

*^a^* Calculated for five-coordinate complexes [58].

**Table 2:** Carboxylate bands [cm^−1^] in the IR spectra of **1**–**11**.

Compound	*ν*_as_(COO^−^)	*ν*_s_(COO^−^)	*∆ ^a^*
[Cu(quin)_2_(H_2_O)]	1631	1372, 1344	287
[Cu(quin)_2_(CH_3_OH)]∙CH_3_OH (**1**)	1645	1380, 1371, 1364, 1346	299
[Cu(quin)_2_(pyro)_2_] (**2**)	1620	1363, 1321	299
(pyroH)[Cu(quin)_2_Cl] (**3**)	1622, 1615	1374, 1365, 1357, 1344	278
[Cu(quin)_2_(morph)_2_] (**4**)	1618	1377, 1363	255
[Cu(quin)_2_(pipe)] (**6**)	1676, 1651	1355, 1341	335
[Cu(quin)_2_(pipe)_2_] (**7**)	1626	1364, 1358, 1346	280
[Cu(quin)_2_(pipe)_2_]∙CH_3_CN (**8**)	1626	1355, 1344	282
[Cu(quin)_2_(pipe)_2_]∙CH_3_CH_2_CN (**9**)	1624	1362, 1345	279
(pipeH)[Cu(quin)_2_Cl] (**11**)	1611	1367, 1355, 1345	266

*^a^* Calculated as *ν*_as_(COO^−^) − *ν*_s_(COO^−^).

**Table 3:** Crystallographic data for **1** to **6**.

	1	2	3	4	5	6
**Empirical formula**	C_22_H_20_CuN_2_O_6_	C_28_H_30_CuN_4_O_4_	C_24_H_22_ClCuN_3_O_4_	C_28_H_30_CuN_4_O_6_	C_14_H_36_CuN_6_O_8_	C_25_H_23_CuN_3_O_4_
**Formula weight**	471.94	550.10	515.43	582.10	480.03	493.00
**Crystal system**	triclinic	monoclinic	triclinic	monoclinic	triclinic	monoclinic
**Space group**	*P* −1	*P* 2_1_/*n*	*P* −1	*P* 2_1_/*n*	*P* −1	*P* 2_1_/*c*
***T* [K]**	150.0	150.00(10)	150.00(10)	150.00(10)	150.00(10)	150.00(10)
***λ* [Å]**	0.71073	0.71073	0.71073	0.71073	0.71073	0.71073
***a* [Å]**	7.0319(2)	10.7782(5)	9.2917(3)	10.8070(6)	6.1497(4)	13.6876(7)
***b* [Å]**	10.7743(2)	17.4803(8)	14.8463(6)	17.7249(13)	6.9128(5)	10.0952(4)
***c* [Å]**	14.0575(5)	13.4856(6)	16.5238(6)	13.4503(7)	14.5118(8)	16.4802(9)
***α* [°]**	105.630(3)	90	102.208(3)	90	80.946(5)	90
***β* [°]**	104.177(3)	91.801(4)	99.808(3)	91.991(5)	89.732(5)	106.995(5)
***γ* [°]**	93.338(3)	90	92.838(3)	90	65.307(7)	90
***V* [Å^3^]**	985.59(5)	2539.5(2)	2186.63(14)	2574.9(3)	552.24(7)	2177.77(19)
***Z***	2	4	4	4	1	4
***D*_calc_ [g/cm^3^]**	1.590	1.439	1.566	1.502	1.443	1.504
***μ* [mm^−1^]**	1.153	0.902	1.159	0.900	1.040	1.042
**Collected reflections**	16,837	15,440	19,241	15,411	9645	20,410
**Unique reflections**	4520	6814	10,079	6603	2983	5898
**Observed reflections**	3968	4457	7902	3330	2614	5007
***R*_int_**	0.0329	0.0399	0.0293	0.0725	0.0539	0.0352
***R*_1_ (*I* > 2*σ*(*I*))**	0.0297	0.0420	0.0339	0.0563	0.0371	0.0332
***wR*_2_ (all data)**	0.0733	0.1117	0.0793	0.1274	0.0858	0.0901

**Table 4:** Crystallographic data for **7** to **11**.

	7	8	9	10	11
**Empirical formula**	C_30_H_34_CuN_4_O_4_	C_32_H_37_CuN_5_O_4_	C_33_H_39_CuN_5_O_4_	C_25_H_44_N_4_	C_25_H_24_ClCuN_3_O_4_
**Formula weight**	578.15	619.20	633.23	400.64	529.46
**Crystal system**	monoclinic	monoclinic	monoclinic	triclinic	triclinic
**Space group**	*P* 2_1_/*c*	*P* 2_1_/*n*	*P* 2_1_/*n*	*P* −1	*P* −1
***T* [K]**	150.00(10)	150.00(10)	150.00(10)	150.00(10)	150.00(10)
***λ* [Å]**	1.54184	1.54184	0.71073	0.71073	1.54184
***a* [Å]**	10.9999(2)	11.8229(2)	14.0265(6)	9.6952(5)	9.2658(2)
***b* [Å]**	13.6491(2)	7.60560(10)	7.5246(4)	11.4886(6)	14.8807(3)
***c* [Å]**	19.0017(3)	16.2266(3)	14.4151(7)	12.0529(8)	17.1665(5)
***α* [°]**	90	90	90	64.013(6)	101.324(2)
***β* [°]**	103.652(2)	90.451(2)	90.172(4)	75.608(5)	99.732(2)
***γ* [°]**	90	90	90	89.605(4)	90.224(2)
***V* [Å^3^]**	2772.29(8)	1459.05(4)	1521.42(13)	1160.85(13)	2285.78(10)
***Z***	4	2	2	2	4
***D*_calc_ [g/cm^3^]**	1.385	1.316	1.382	1.146	1.539
***μ* [mm^−1^]**	1.457	1.384	0.764	0.068	2.749
**Collected reflections**	14,885	6018	7848	14,513	28,590
**Unique reflections**	5618	2937	3985	5341	9361
**Observed reflections**	4711	2749	3394	3465	8533
***R*_int_**	0.0376	0.0206	0.0202	0.0394	0.0267
***R*_1_ (*I* > 2*σ*(*I*))**	0.0419	0.0356	0.0315	0.0573	0.0297
***wR*_2_ (all data)**	0.1237	0.0951	0.0854	0.1536	0.0799

## Supplementary Materials

Figure S1: Identification of the aged [Cu(quin)_2_(CH_3_OH)]∙CH_3_OH (**1**). Figure S2: TG and DSC curves for [Cu(quin)_2_(CH_3_OH)]∙CH_3_OH (**1**). Table S1: *π*⋯*π* stacking interactions in [Cu(quin)_2_(CH_3_OH)]∙CH_3_OH (**1**). Figure S3: Overlay of the [Cu(quin)_2_(pyro)_2_] molecules in **2**. Table S2: Overlay parameters for pairs of complex molecules in **2**, **4**, and **7**. Figure S4: Chains in the structures of [Cu(quin)_2_(morph)_2_] (**4**) and [Cu(quin)_2_(pipe)_2_] (**7**). Figure S5: A view along the chains in the structure of [Cu(quin)_2_(pyro)_2_] (**2**). Figure S6: Packing in the structure of [Cu(quin)_2_(pipe)] (**6**). Table S3: Overlay parameters for the [Cu(quin)_2_(pipe)_2_] complex molecules in **7**, **8**, and **9**. Figure S7: Packing motifs in [Cu(quin)_2_(pipe)_2_] (**7**) and [Cu(quin)_2_(pipe)_2_]∙CH_3_CN (**8**). Figure S8: A view along the channels in the structure of [Cu(quin)_2_(pipe)_2_]∙CH_3_CN (**8**). Table S4: Intermolecular interactions in [Cu(quin)_2_(pipe)] (**6**). Figure S9: Hydrogen bonding pattern in (pyroH)[Cu(quin)_2_Cl] (**3**). Figure S10: Hydrogen bonds in [Cu(en)_2_(H_2_O)_2_](morphCOO)_2_ (**5**). Figure S11: Packing arrangement in the structure of **10**. Table S5: Hydrogen bonds in compounds **1**–**11**. Figure S12: Infrared spectrum of [Cu(quin)_2_(H_2_O)]. Figures S13–S21: Infrared spectra of **1**–**4**, **6**–**9** and **11**.
