# Supplementary material for: Beyond the Simple Copper(II) Coordination Chemistry with Quinaldinate and Secondary Amines
Source: Molecules. 2020 Mar 30;25(7):1573. doi: 10.3390/molecules25071573 (PMC7180772; doi:10.3390/molecules25071573)
Supplement: Supplementary file 1 [file molecules-25-01573-s001.zip › Supplementary materials.pdf]

Supplementary material

for

Beyond the simple copper(II) coordination chemistry with quinaldinate and secondary amines

Barbara Modec *, Nina Podjed * and Nina Lah #

Department of Chemistry and Chemical Technology, University of Ljubljana,
Večna pot 113, 1000 Ljubljana, Slovenia.
# Current address: Lek Pharmaceuticals, Ljubljana, Slovenia. E-mail: nina.lah@novartis.com

* Corresponding authors. E-mail: barbara.modec@fkkt.uni-lj.si, nina.podjed@fkkt.uni-lj.si

Figure S1. Identification of the aged [Cu(quin)$_2$(CH$_3$OH)]·CH$_3$OH (**1**): (a) measured PXRD, (b) calculated pattern for [Cu(quin)$_2$(H$_2$O)] [S1], and (c) calculated pattern for the new polymorph of [Cu(quin)$_2$(H$_2$O)].

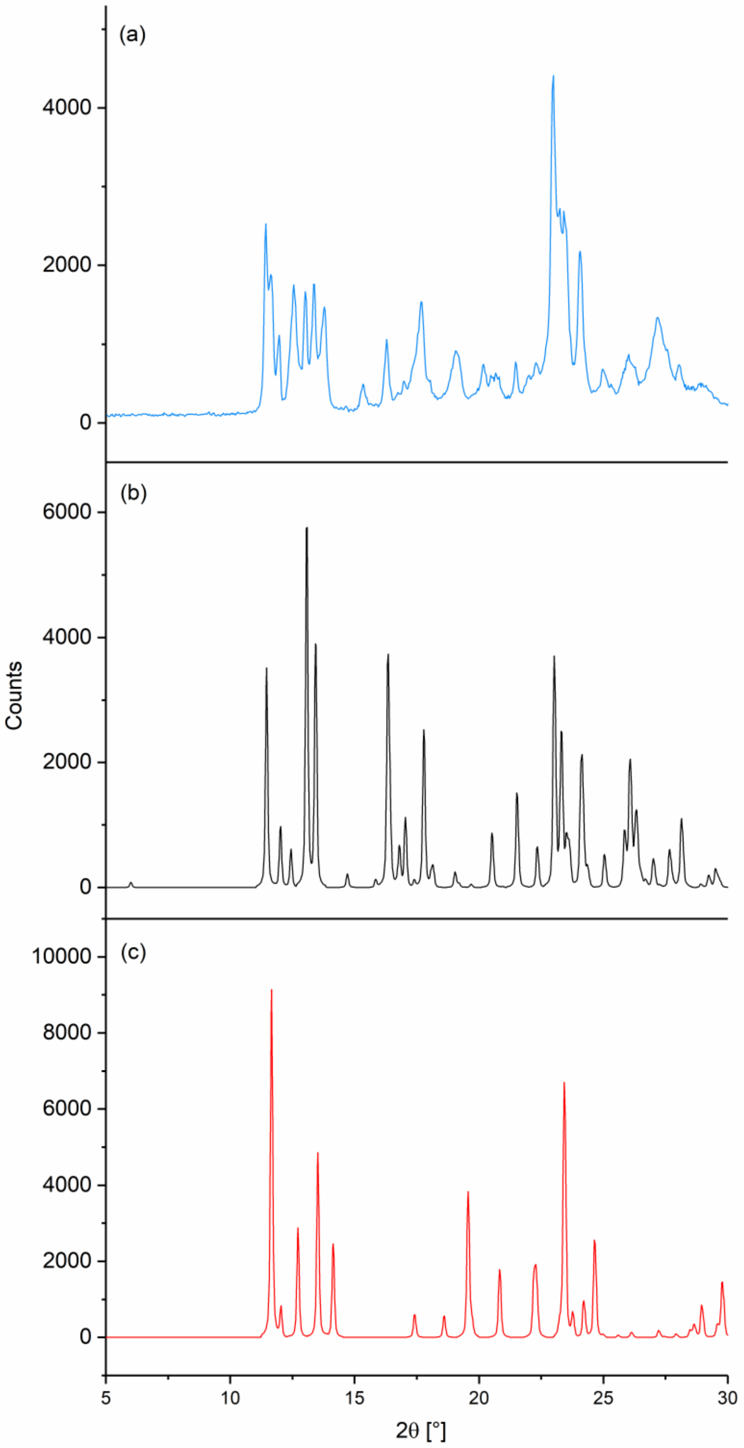

Figure S2. TG and DSC curves for [Cu(quin)$_2$(CH$_3$OH)]·CH$_3$OH (**1**).

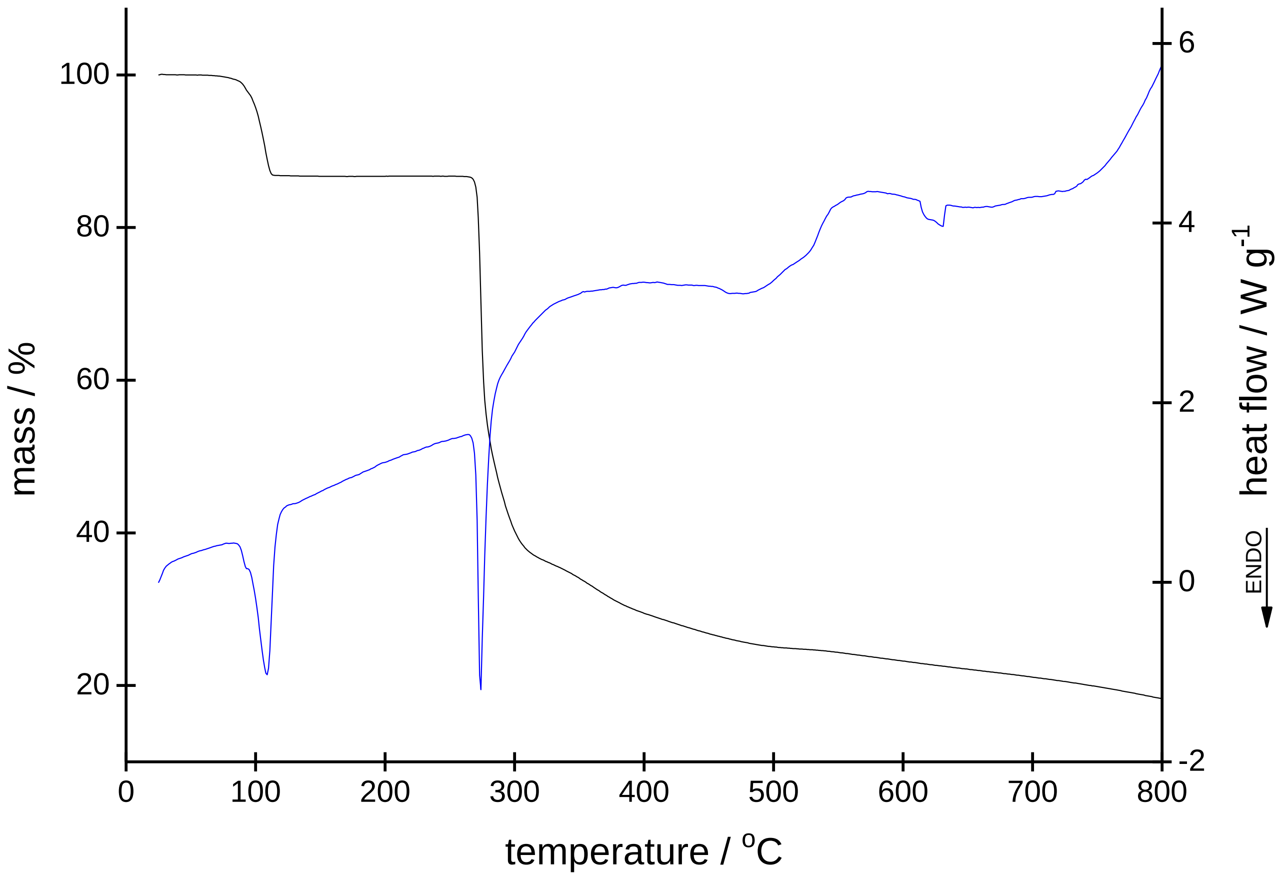

A list of $\pi\cdots\pi$ stacking interactions in [Cu(quin)$_2$(CH$_3$OH)]·CH$_3$OH (1).

The labels *Cg*(3) and *Cg*(4) pertain to the centroids of the pyridine parts of the quinaldinates, *i.e.,* N(1)-C(11)-C(12)-C(13)-C(14)-C(19) and N(2)-C(21)-C(22)-C(23)-C(24)-C(29), respectively, and the label *Cg*(6) to the arene part of one quinaldinate, *i.e.,* C(24)-C(25)-C(26)-C(27)-C(28)-C(29). Parameters *Cg*···*Cg*, interplanar distance, dihedral and offset angles are as defined in reference [S2].

Table S1. $\pi\cdots\pi$ stacking interactions [Å, °] in [Cu(quin)$_2$(CH$_3$OH)]·CH$_3$OH (**1**).

$\pi\cdots\pi$ interactions	type	*Cg*···*Cg*	interplanar distance	dihedral angle	offset angle
Cg(4)···*Cg*(4) [1–*x*, 1–*y*, 2–*z*]	pyridine···pyridine	3.6148(11)	3.3167(8)	0.02(10)	23.4
Cg(3)···*Cg*(6) [–*x*,–*y*,2–*z*]	pyridine···arene	4.0098(12)	2.9564(9)	20.83(10)	23.1

Figure S3. Overlay of the [Cu(quin)$_2$(pyro)$_2$] molecules in **2**, done by Mercury [S3].

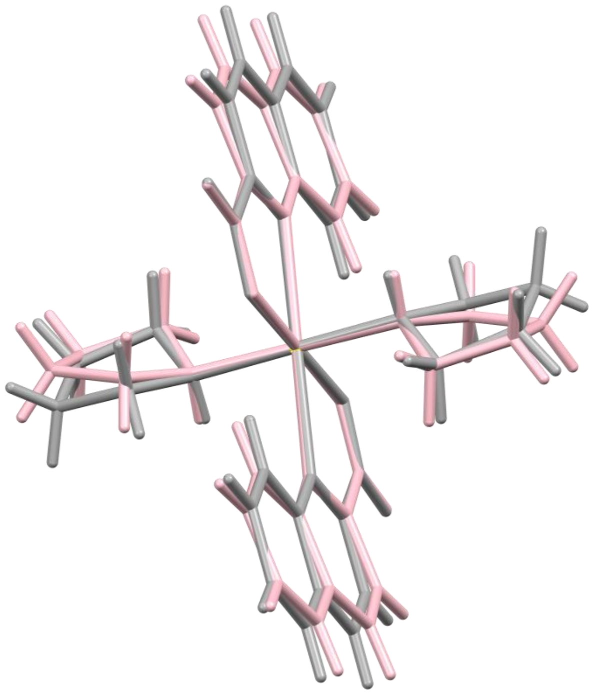

Table S2. Overlay parameters for pairs of complex molecules in **2**, **4** and **7**.[a]

Compound	RMSD[b]	Max. distance[c]
**2**	0.2082	0.6469
**4**	0.2270	0.4844
**7**	0.1594	0.2959

[a] Overlay was done by Mercury [S3].

[b] Root-mean-square deviation.

[c] Maximum distance in Å between two equivalent atoms in the overlaid molecules.

Figure S4. Chains in the structures of [Cu(quin)$_2$(morph)$_2$] (**4**) (top) and [Cu(quin)$_2$(pipe)$_2$] (**7**) (bottom).

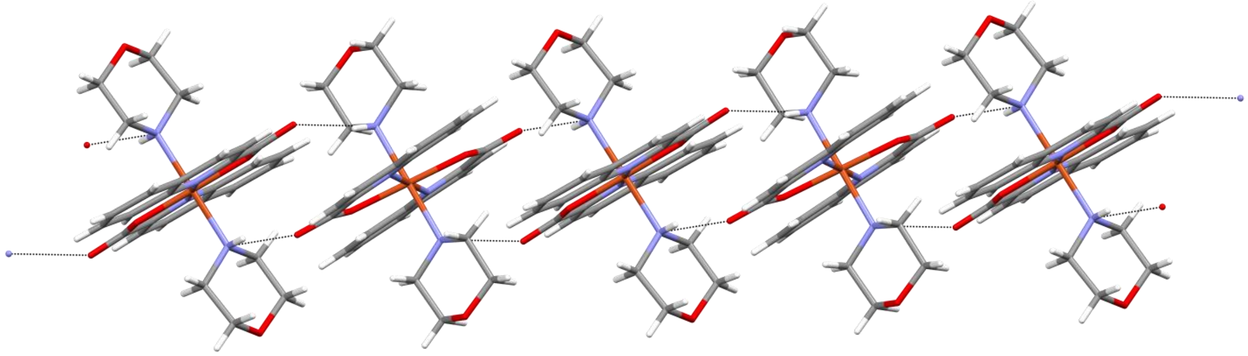

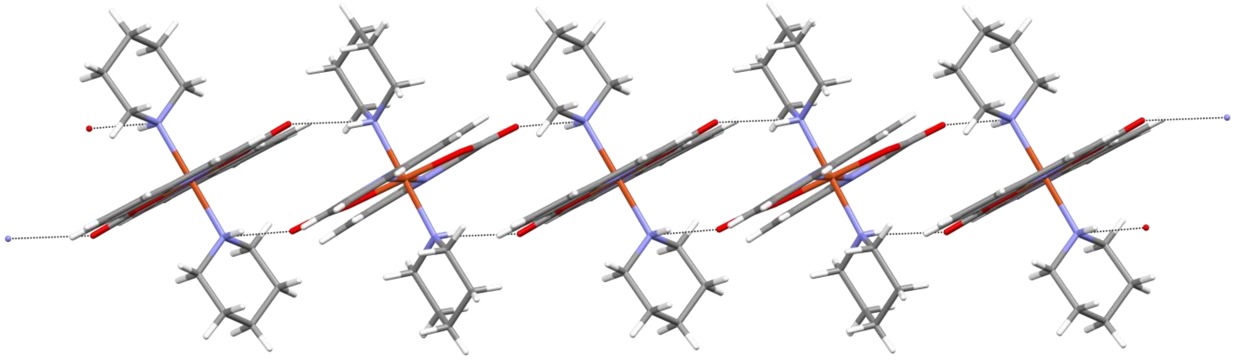

Figure S5. A view along the chains in the structure of [Cu(quin)$_2$(pyro)$_2$] (**2**). The chains pack in a compact manner: each chain is surrounded by six adjacent ones.

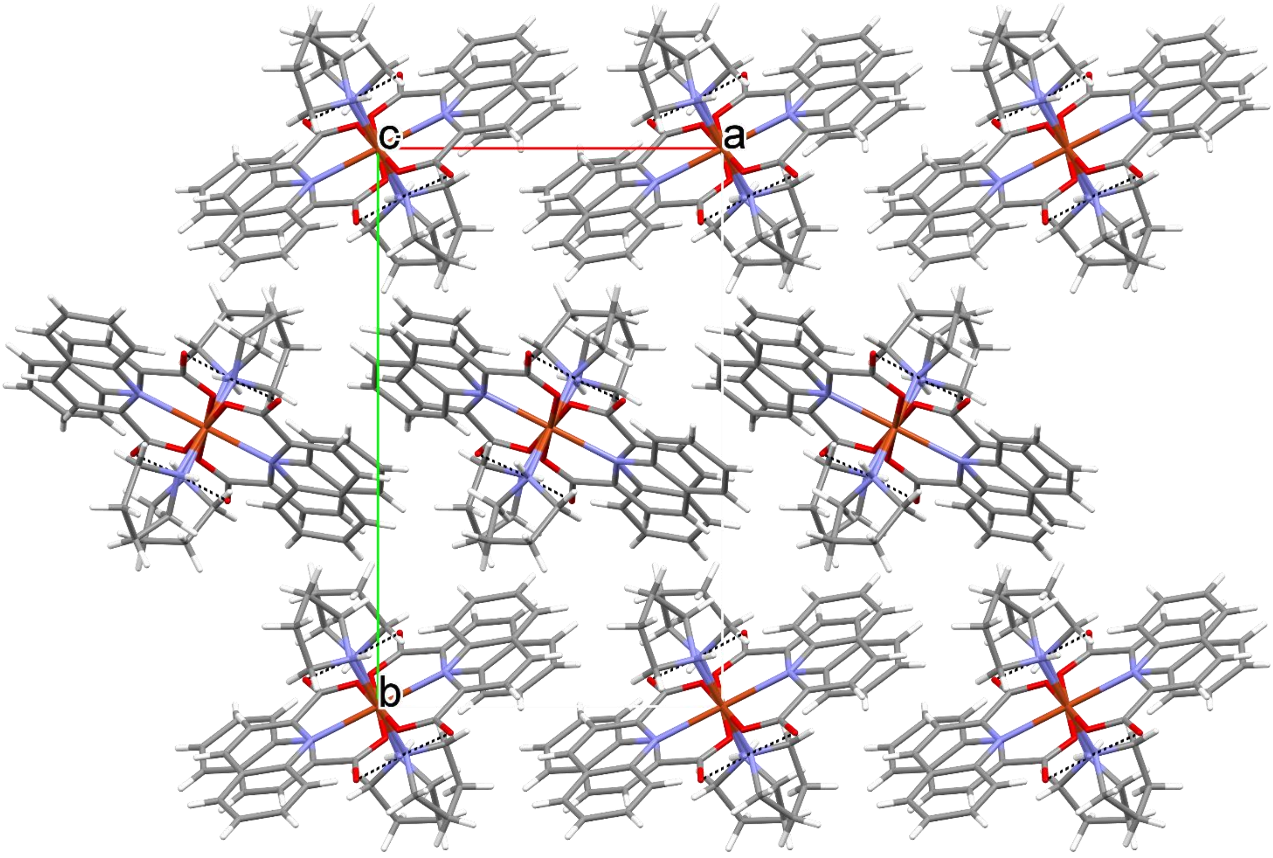

Figure S6. Packing in the structure of [Cu(quin)$_2$(pipe)] (**6**). Complex molecules are held together with the agency of $\pi\cdots\pi$ stacking interactions between pairs of quinaldinate rings.

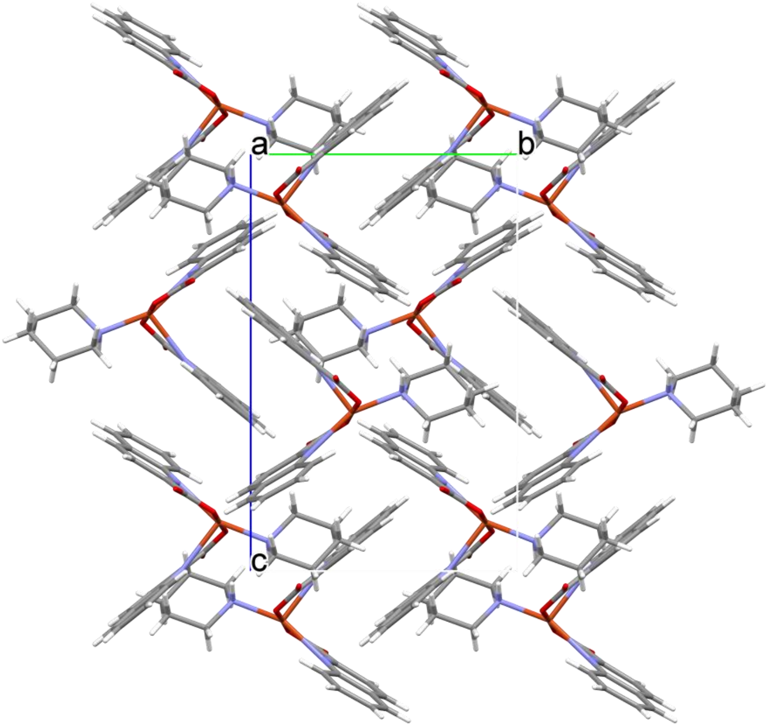

Table S3. Overlay parameters for the $[Cu(quin)_2(pipe)_2]$ complex molecules in **7**, **8** and **9**.[a]

Pairs of compounds	RMSD[b]	Max. distance[c]
**7** and **8**[d]	0.1836	0.3717
	0.2458	0.4394
**7** and **9**[d]	0.2452	0.4392
	0.2962	0.5059
**8** and **9**	0.0690	0.1281

[a] Overlay was done by Mercury [S3].

[b] Root-mean-square deviation.

[c] Maximum distance in Å between two equivalent atoms in the overlaid molecules.

[d] As there are two complex molecules in the asymmetric unit of **7**, two sets of parameters are given.

Figure S7. Packing motifs in [Cu(quin)$_2$(pipe)$_2$] (**7**) (top) and [Cu(quin)$_2$(pipe)$_2$]·CH_3CN (**8**) (bottom). Both views are along the chains. Please note that [Cu(quin)$_2$(pipe)$_2$]·CH_3CN (**8**) and [Cu(quin)$_2$(pipe)$_2$]·CH_3CH_2CN (**9**) are isomorphous. The packing of chains in **8** and **9** creates hydrophobic channels.

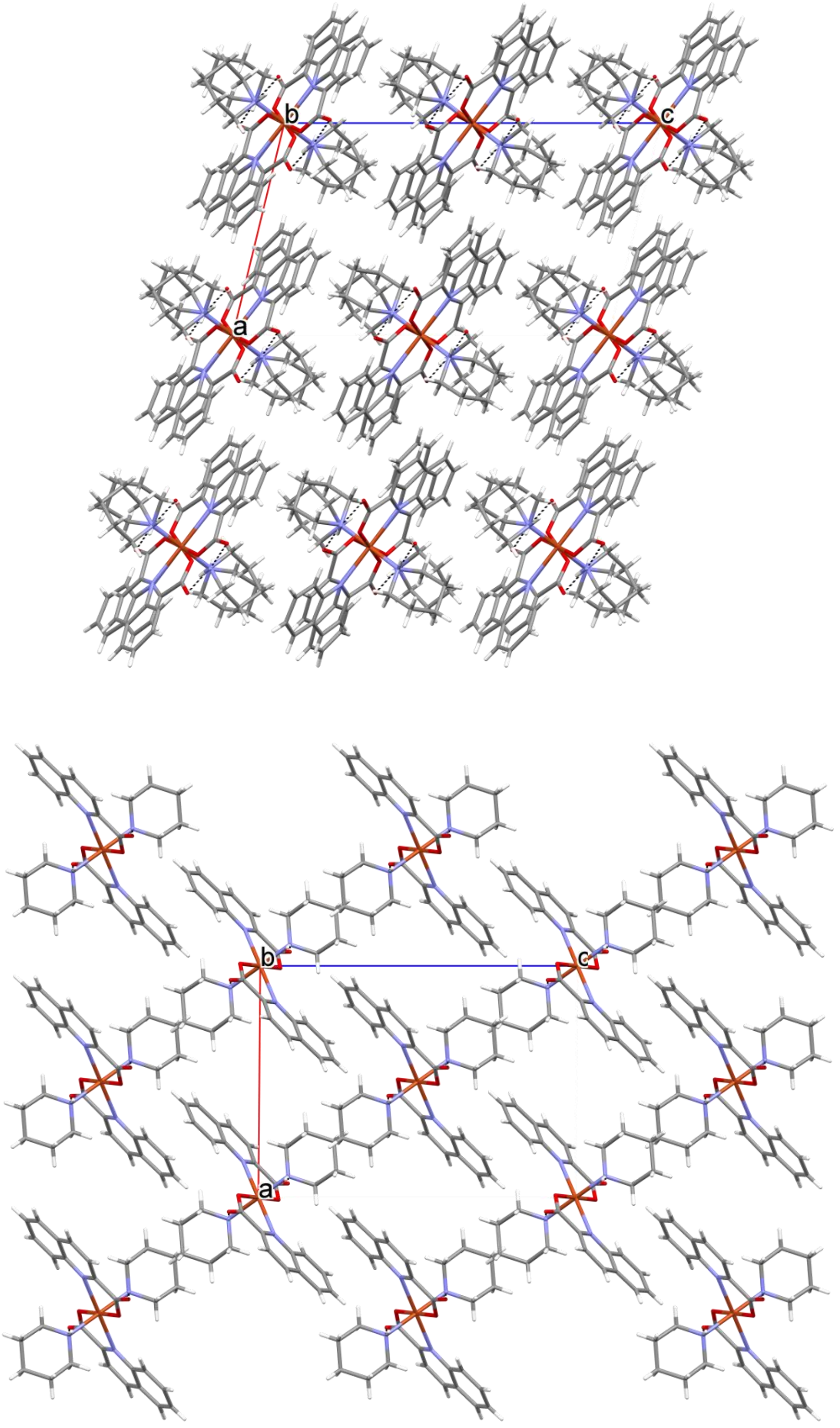

Figure S8. A view along the channels, shown in red colour, in the structure of $[Cu(quin)_2(pipe)_2]\cdot CH_3CN$ (**8**). Solvent molecules of crystallization that are accommodated in the channels are not shown.

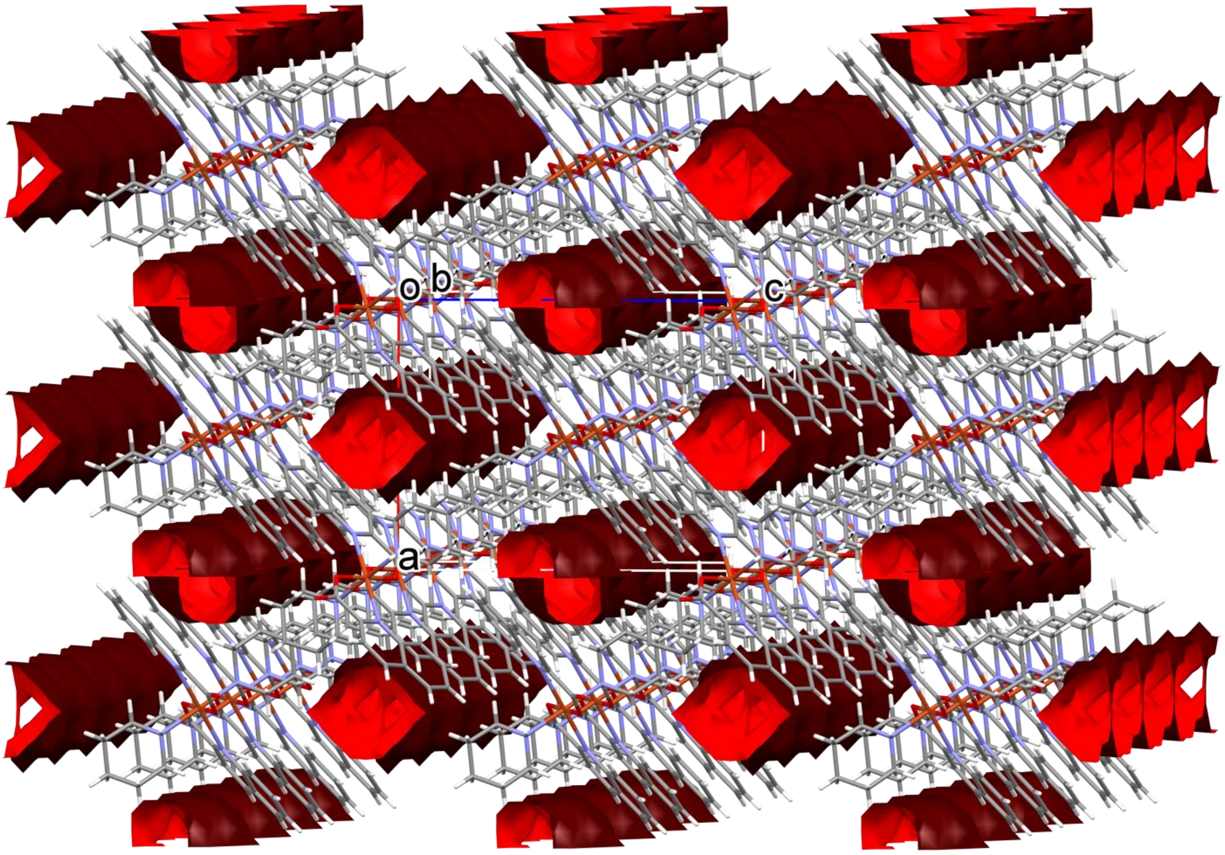

A list of intermolecular interactions in [Cu(quin)$_2$(pipe)] (6).

The labels *Cg*(3) and *Cg*(4) pertain to the centroids of the pyridine parts of the quinaldinates, *i.e.,* N(1)-C(11)-C(12)-C(13)-C(14)-C(19) and N(2)-C(21)-C(22)-C(23)-C(24)-C(29), respectively, and the labels *Cg*(6) and *Cg*(7) to the corresponding arene parts, *i.e.,* C(14)-C(15)-C(16)-C(17)-C(18)-C(19) and C(24)-C(25)-C(26)-C(27)-C(28)-C(29), respectively. Parameters *Cg*···*Cg*, interplanar distance, dihedral and offset angles are as defined in reference [S2].

Table S4. Intermolecular interactions [Å, °] in [Cu(quin)$_2$(pipe)] (**6**).

**π···π interactions**	**type**	***Cg*···*Cg***	**interplanar distance**	**dihedral angle**	**offset angle**
Cg(4)···*Cg*(6) [*x*, 1/2–*y*, –1/2+*z*]	pyridine···arene	3.7087(10)	3.5616(7)	11.77(8)	27.2
Cg(6)···*Cg*(7) [*x*, 1/2–*y*, 1/2+*z*]	arene···arene	3.9004(10)	3.4550(7)	11.41(8)	23.9
**C–H···π interactions**	**H···*Cg***	**C–H···*Cg***	**C···*Cg***	**X–H, π**	
C(32)–H···*Cg*(3) [*x*, 1+*y*, *z*]	3.00	159	3.918(2)	67	
C(33)–H···*Cg*(7) [*x*, 1+*y*, *z*]	2.78	146	3.630(2)	60	

Figure S9. Hydrogen bonding pattern in (pyroH)[Cu(quin)$_2$Cl] (**3**): $NH_2^+ \cdots COO^-$ interactions link four pyrrolidinium cations with four complex anions into a cyclic supramolecular motif, described in graph set notation as $R_8^8(36)$ [S4]. Colour code: complex anions - grey, pyrrolidinium cations - gold.

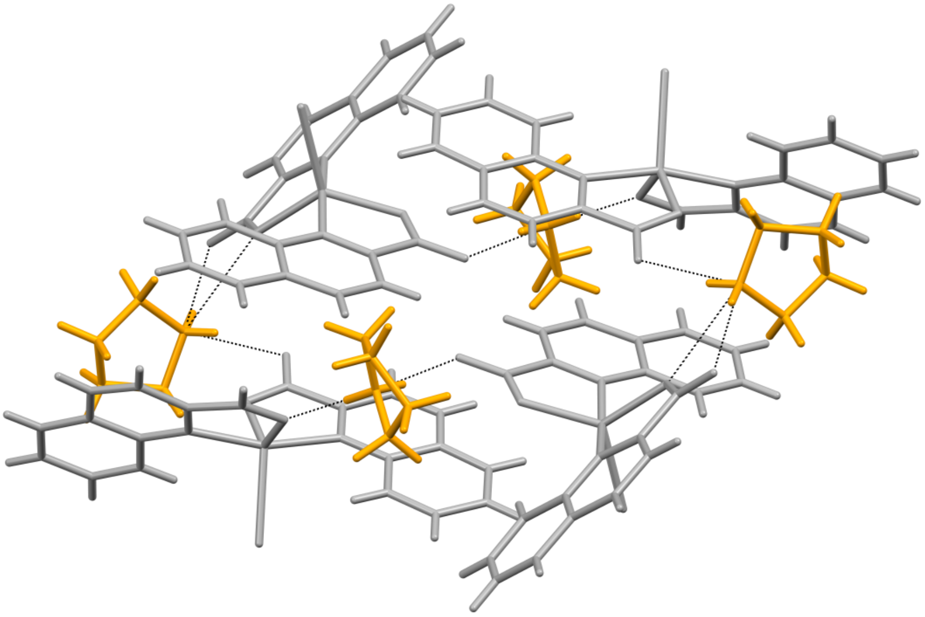

Figure S10. Hydrogen bonds in $[Cu(en)_2(H_2O)_2](morphCOO)_2$ (**5**): intricate pattern of H-bonds (dotted lines) links complex cations (light grey) and counteranions (gold) into infinite 2D array. The surface of the layers is covered with the hydrophobic part of the morphCOO⁻ anions. The layers stack along *c*-axis.

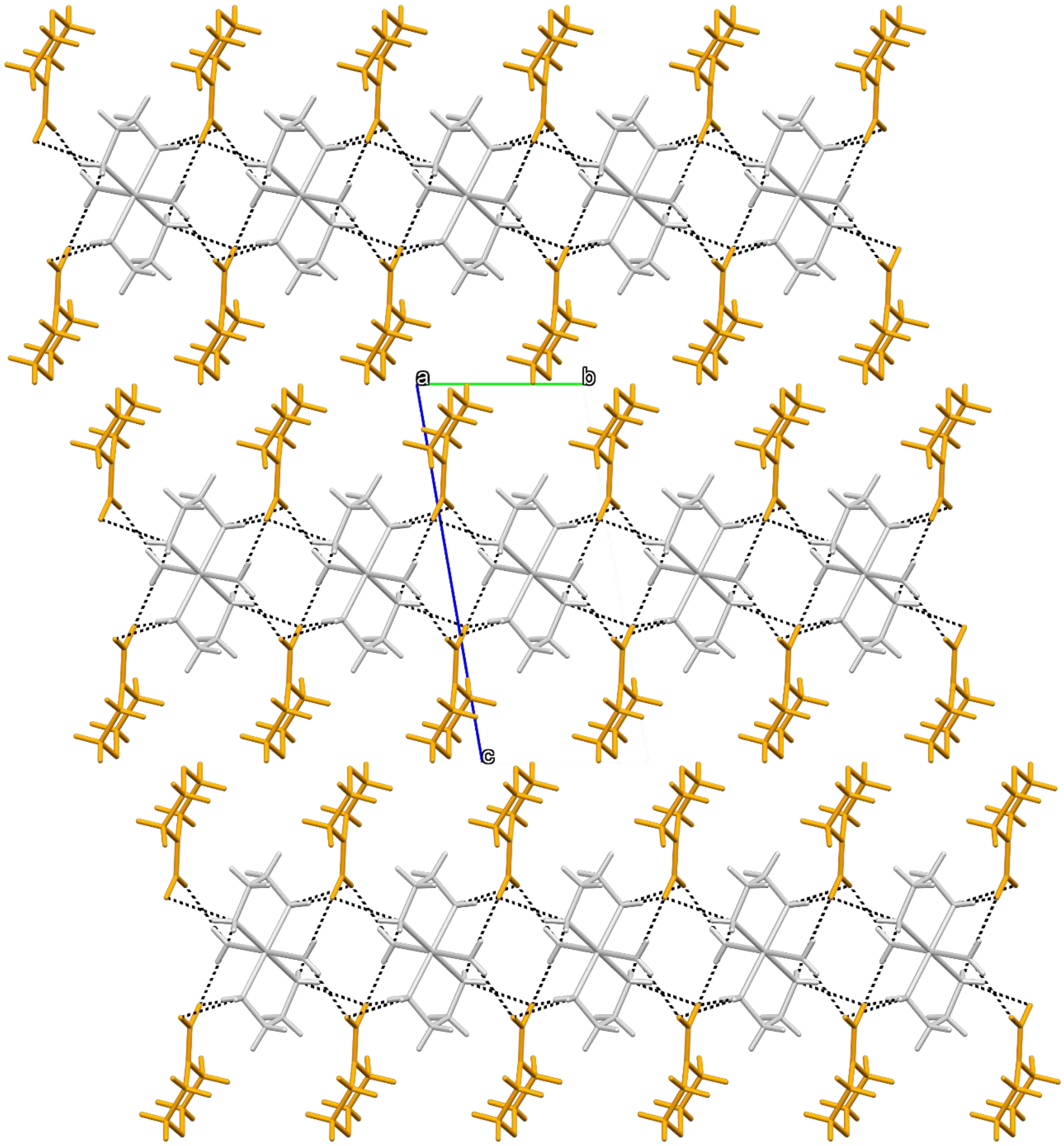

Figure S11. Packing arrangement in the structure of **10**.

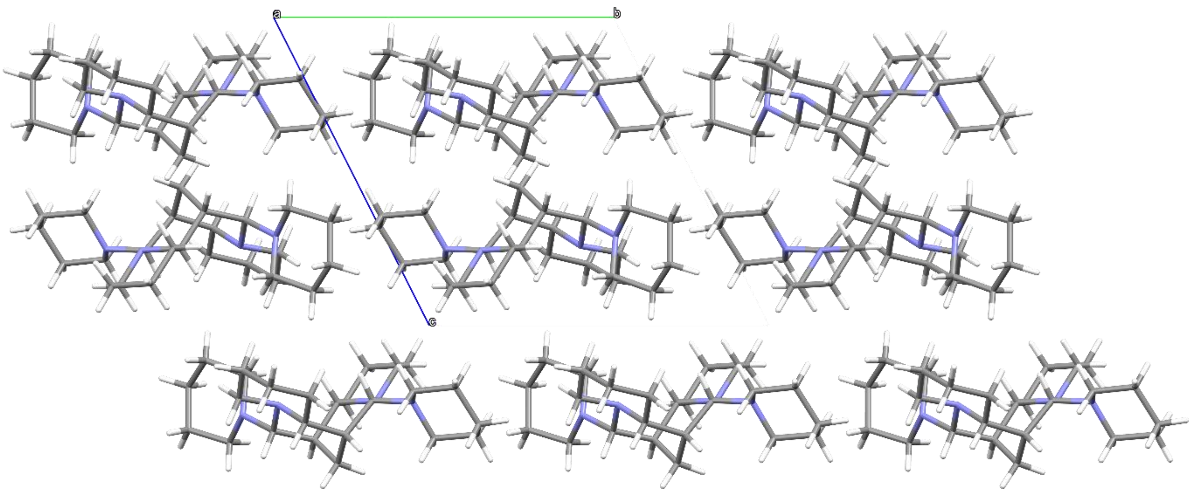

Table S5. Hydrogen bonds in compounds **1–11**.

**Compound**	**Donor···acceptor**	**Description**	**Distance [Å]**
[Cu(quin)$_2$(CH$_3$OH)]·CH$_3$OH (**1**)	O1M···O12[1–*x*, –*y*, 2–*z*]	CH$_3$OH(ligand)···COO$^-$	2.752(2)
	O2M···O22	CH$_3$OH···COO$^-$	2.743(2)
[Cu(quin)$_2$(pyro)$_2$] (**2**)	N3···O22	NH(pyro)···COO$^-$	2.903(3)
	N4···O12[2–*x*, –*y*, 4–*z*]	NH(pyro)···COO$^-$	2.916(2)
(pyroH)[Cu(quin)$_2$Cl] (**3**)	N5···O11	NH$_2^+$(pyroH$^+$)···COO$^-$	2.828(3)
	N5···O32	NH$_2^+$(pyroH$^+$)···COO$^-$	2.730(3)
	N6···O22	NH$_2^+$(pyroH$^+$)···COO$^-$	2.802(3)
	N6···O42[1–*x*, 1–*y*, 1–*z*]	NH$_2^+$(pyroH$^+$)···COO$^-$	2.879(3)
[Cu(quin)$_2$(morph)$_2$] (**4**)	N3···O22	NH(morph)···COO$^-$	2.924(4)
	N4···O12	NH(morph)···COO$^-$	2.952(4)
trans-[Cu(en)$_2$(H$_2$O)$_2$](morphCOO)$_2$ (**5**)	N1···O1[1+*x*, *y*, *z*]	NH$_2$···H$_2$O	3.056(2)
	N1···O31	NH$_2$···COO$^-$	3.001(2)
	N2···O31[*x*, –1+*y*, *z*]	NH$_2$···COO$^-$	2.987(2)
	N2···O32[1+*x*, –1+*y*, *z*]	NH$_2$···COO$^-$	2.907(2)
	O1···O31[–*x*, 2–*y*, 1–*z*]	H$_2$O···COO$^-$	2.7773(19)
	O1···O32	H$_2$O···COO$^-$	2.7447(18)
[Cu(quin)$_2$(pipe)$_2$] (**7**)	N3···O22[2–*x*, 1–*y*, 1–*z*]	NH(pipe)···COO$^-$	2.924(2)
	N4···O12[2–*x*, –*y*, 1–*z*]	NH(pipe)···COO$^-$	2.994(2)
[Cu(quin)$_2$(pipe)$_2$]·CH$_3$CN (**8**)	N2···O12[*x*, –1+*y*, *z*]	NH(pipe)···COO$^-$	3.0103(17)
[Cu(quin)$_2$(pipe)$_2$]·CH$_3$CH$_2$CN (**9**)	N2···O12[*x*, –1+*y*, *z*]	NH(pipe)···COO$^-$	2.9973(17)
(pipeH)[Cu(quin)$_2$Cl] (**11**)	N5···O22[1–*x*, –*y*, 2–*z*]	NH$_2^+$(pipeH$^+$)···COO$^-$	2.8505(18)
	N5···O42[1–*x*, –*y*, 2–*z*]	NH$_2^+$(pipeH$^+$)···COO$^-$	2.8222(18)
	N6···O12	NH$_2^+$(pipeH$^+$)···COO$^-$	2.7886(18)
	N6···O32[–*x*, 1–*y*, 2–*z*]	NH$_2^+$(pipeH$^+$)···COO$^-$	2.8471(18)

Figure S12. Infrared spectrum of [Cu(quin)$_2$(H_2O)].

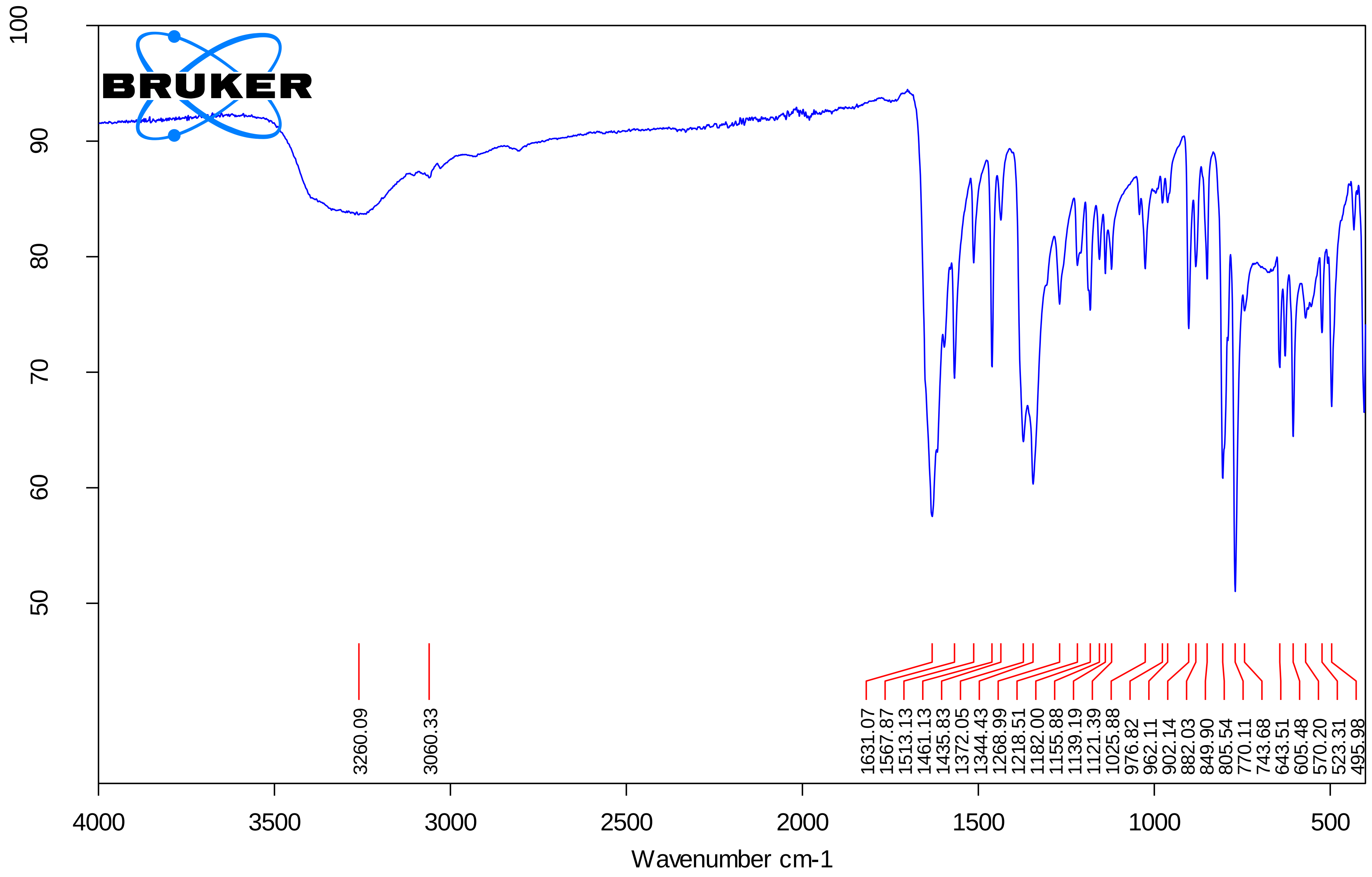

Figure S13. Infrared spectrum of $[Cu(quin)_2(CH_3OH)]\cdot CH_3OH$ (**1**).

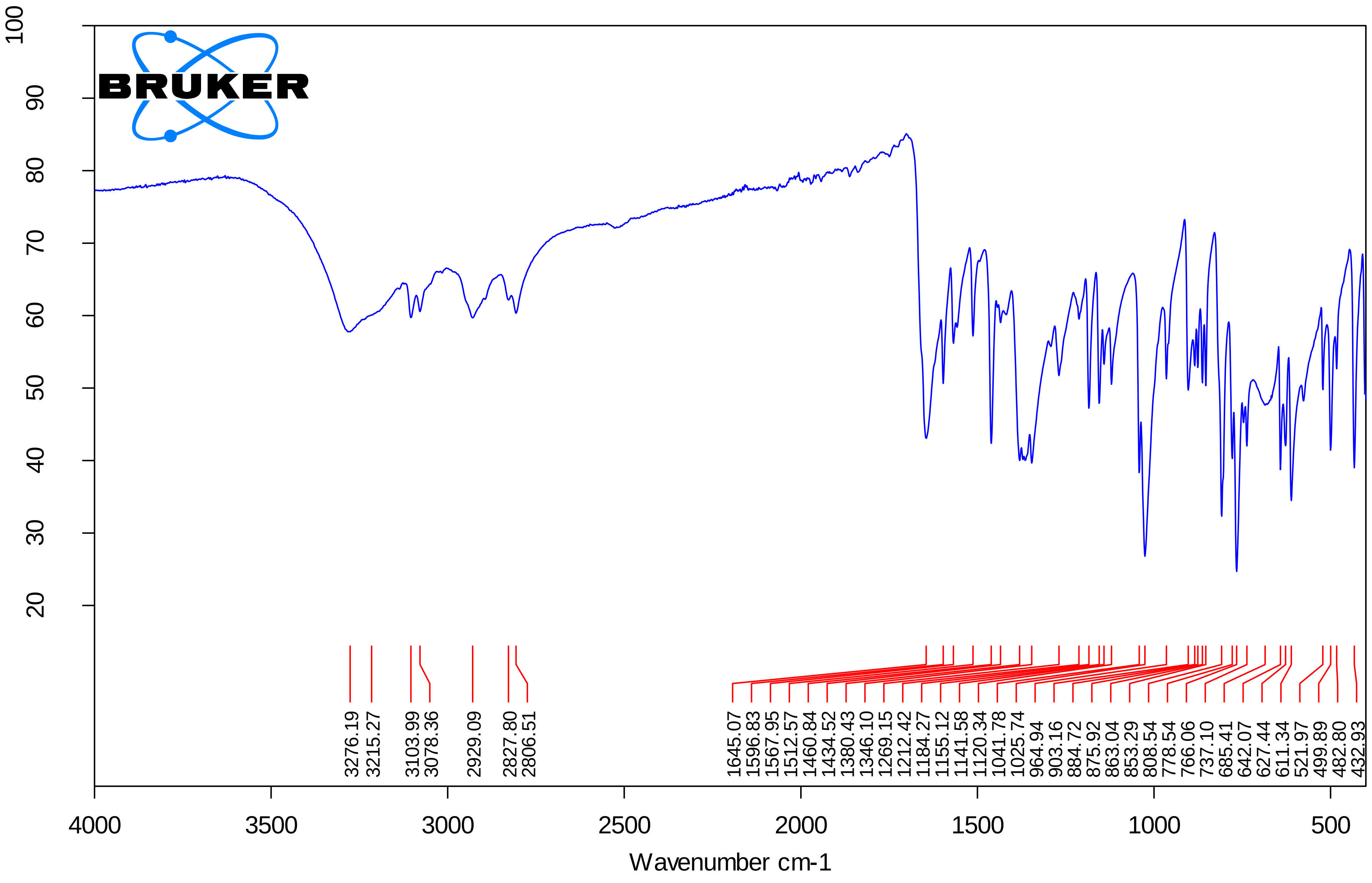

Figure S14. Infrared spectrum of [Cu(quin)$_2$(pyro)$_2$] (**2**).

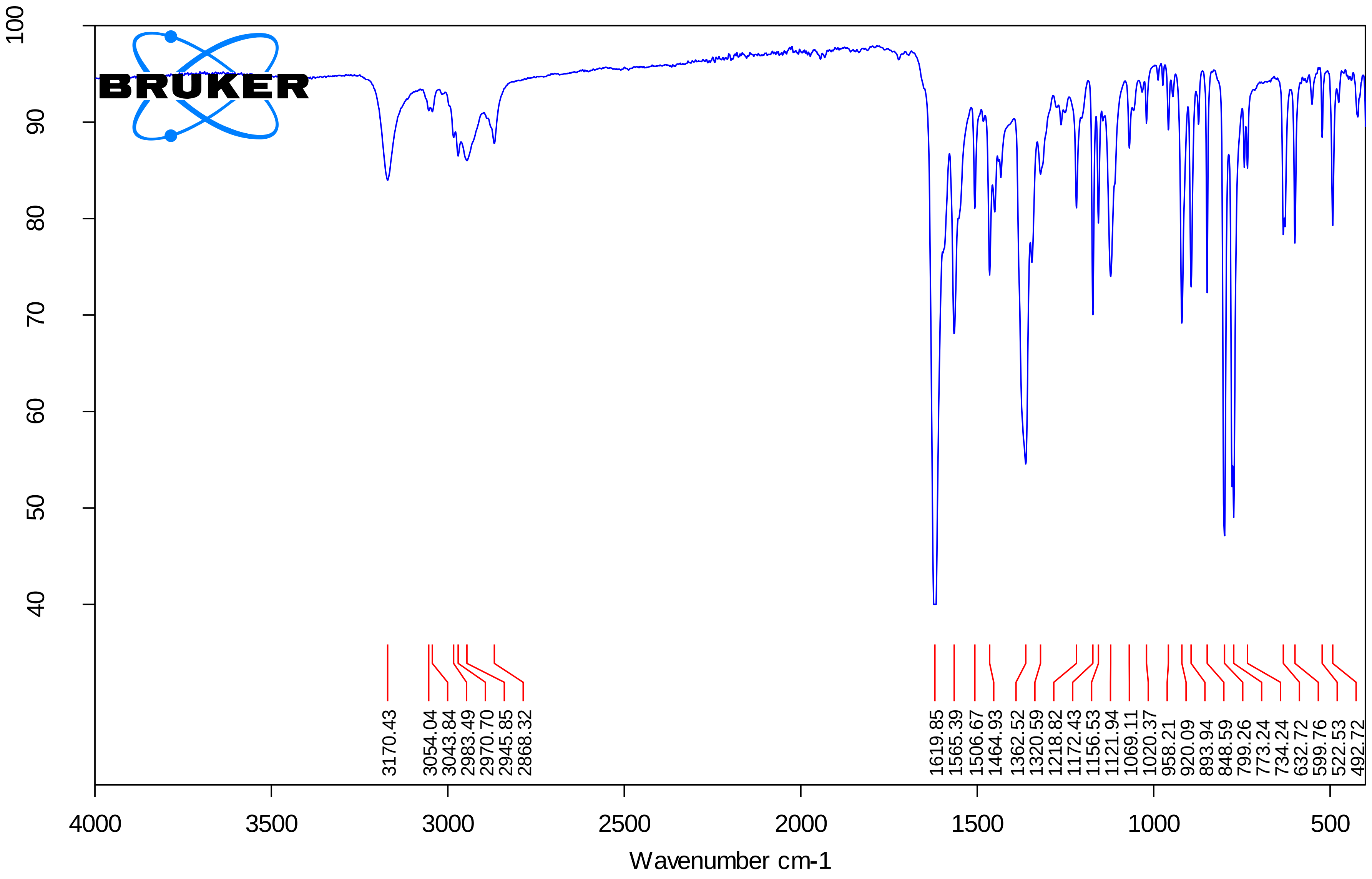

Figure S15. Infrared spectrum of (pyroH)[Cu(quin)$_2$Cl] (**3**).

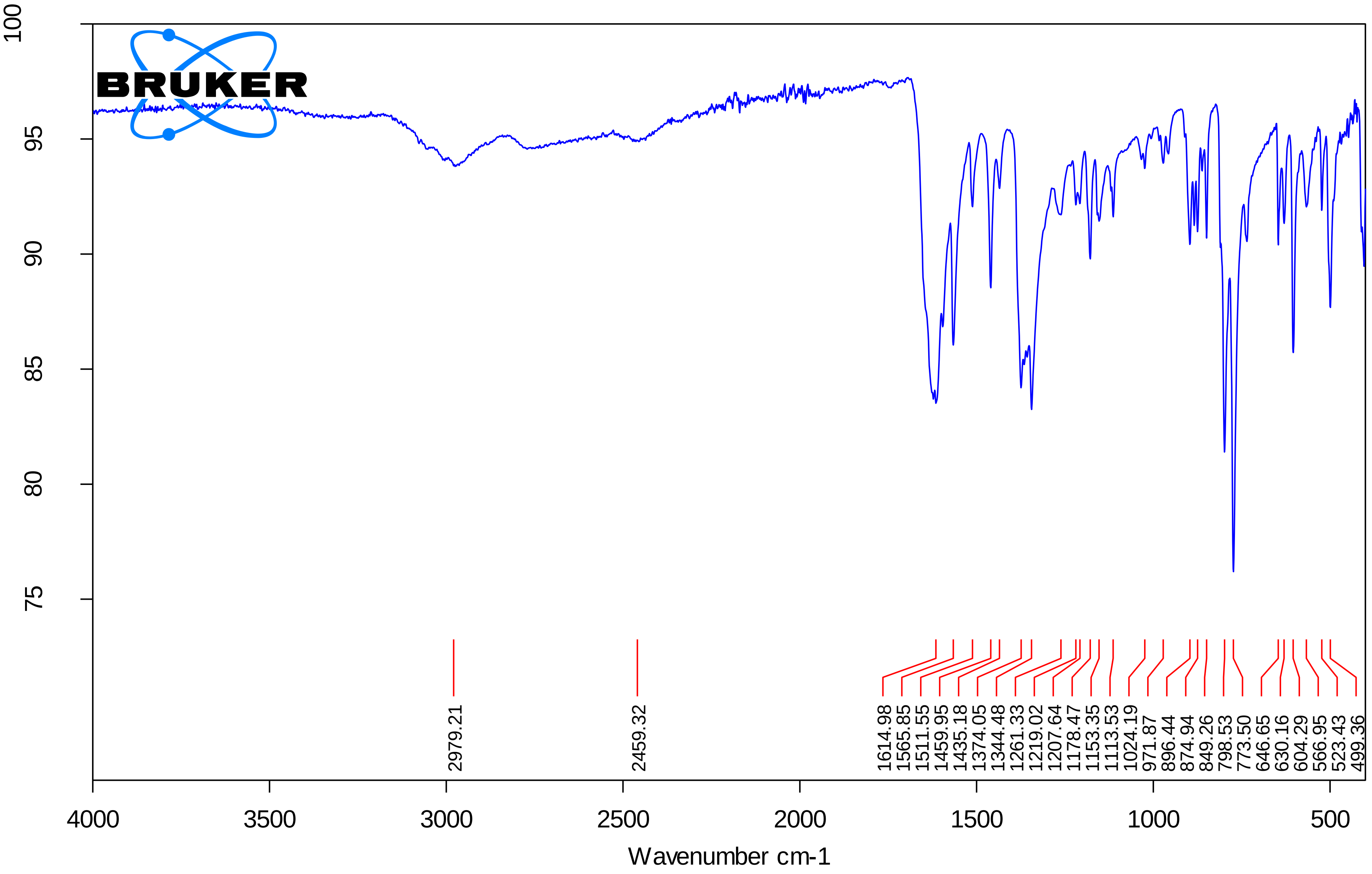

Figure S16. Infrared spectrum of [Cu(quin)$_2$(morph)$_2$] (**4**).

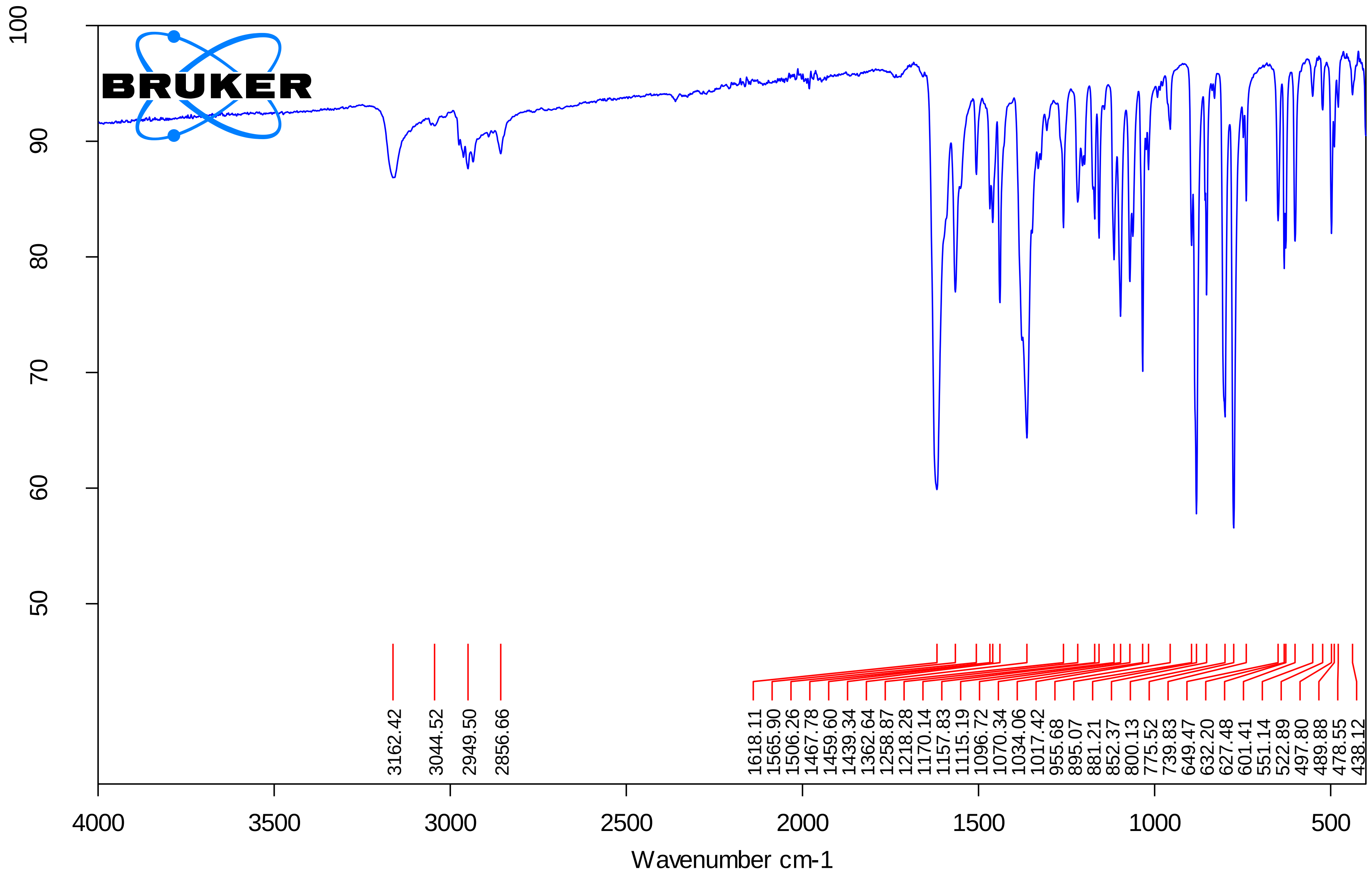

Figure S17. Infrared spectrum of [Cu(quin)$_2$(pipe)] (**6**).

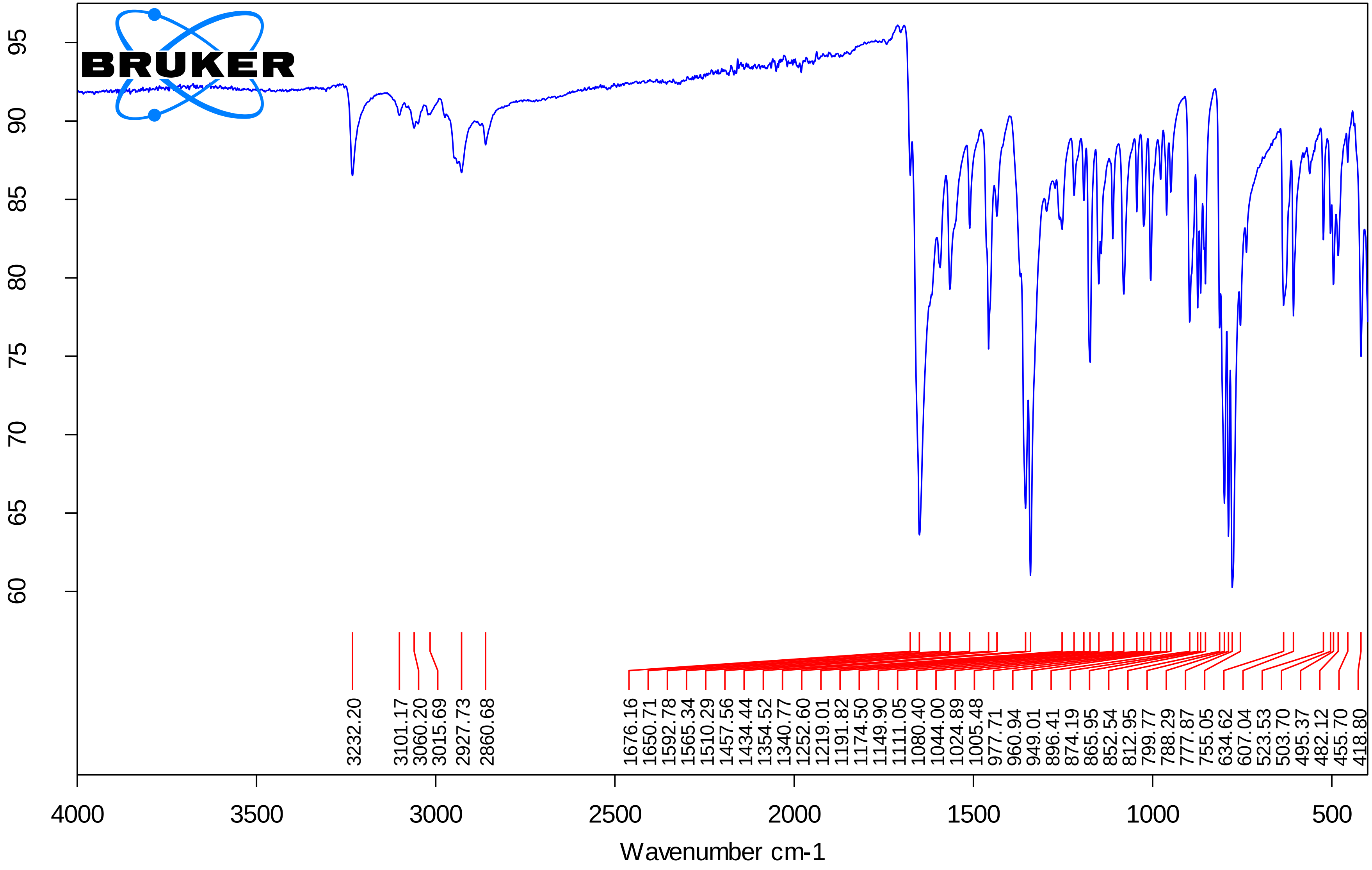

Figure S18. Infrared spectrum of [Cu(quin)$_2$(pipe)$_2$] (**7**).

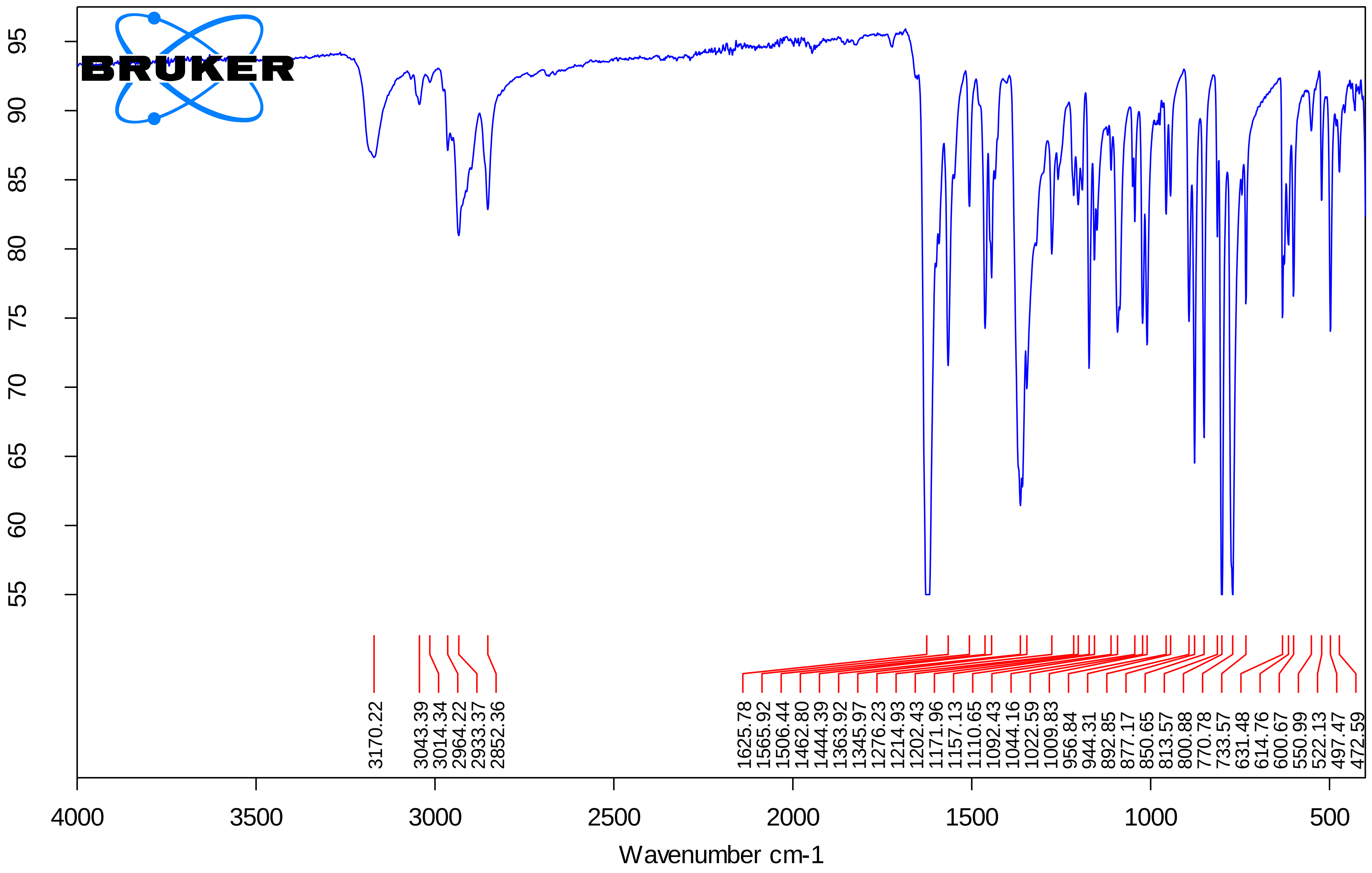

Figure S19. Infrared spectrum of [Cu(quin)$_2$(pipe)$_2$]·CH_3CN (**8**).

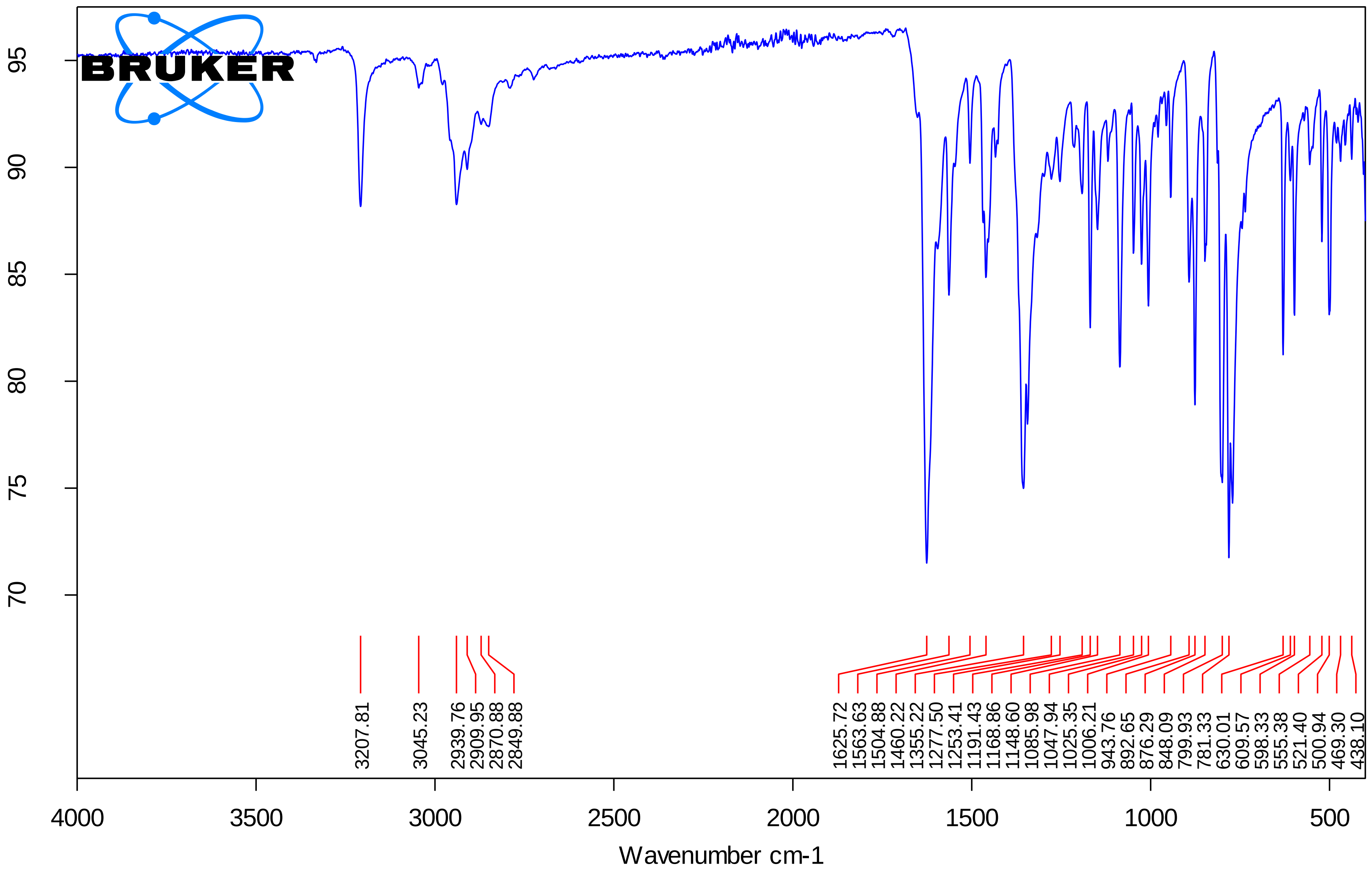

Figure S20. Infrared spectrum of [Cu(quin)$_2$(pipe)$_2$]·CH_3CH_2CN (**9**).

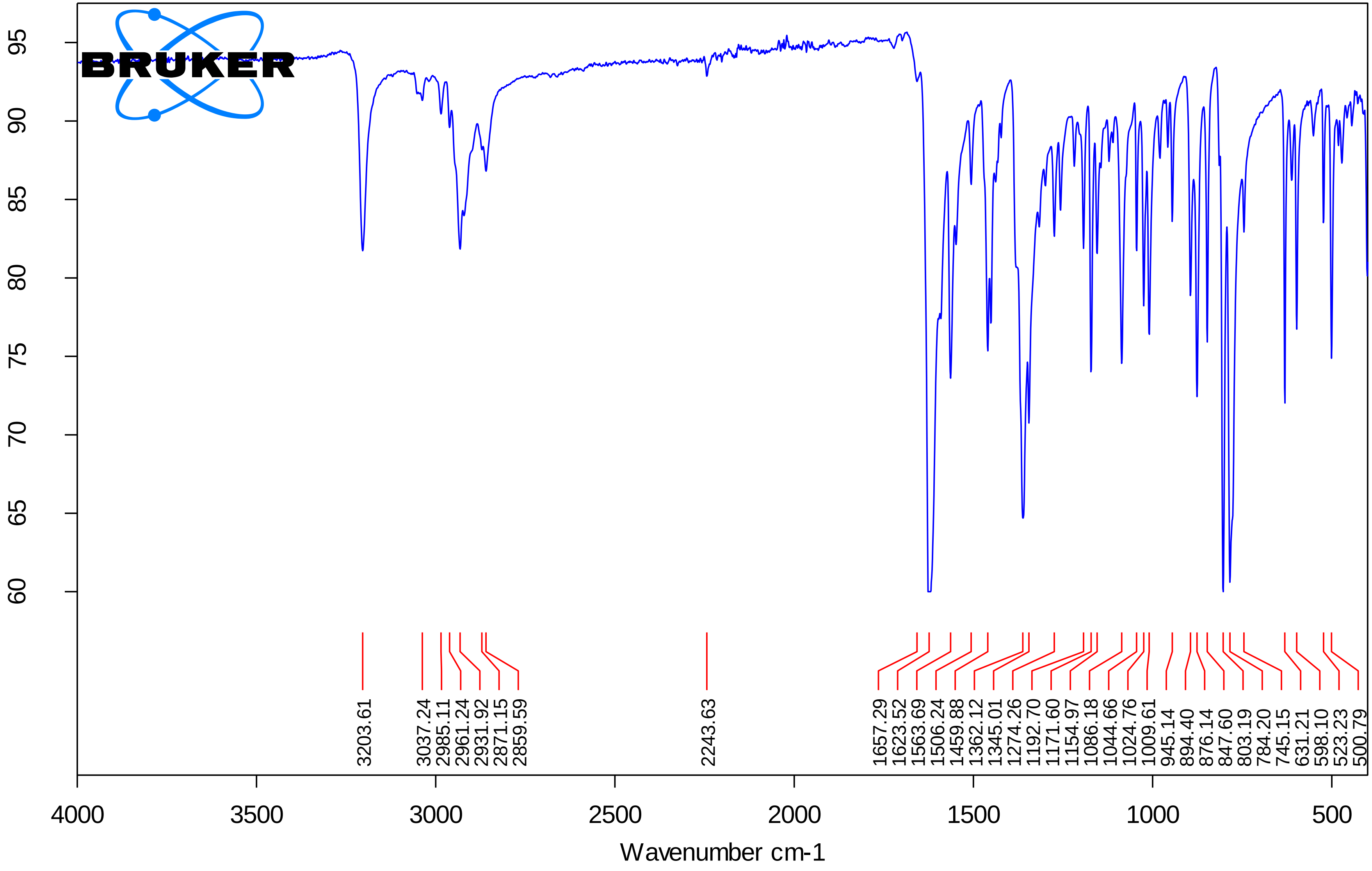

Figure S21. Infrared spectrum of (pipeH)[Cu(quin)$_2$Cl] (**11**).

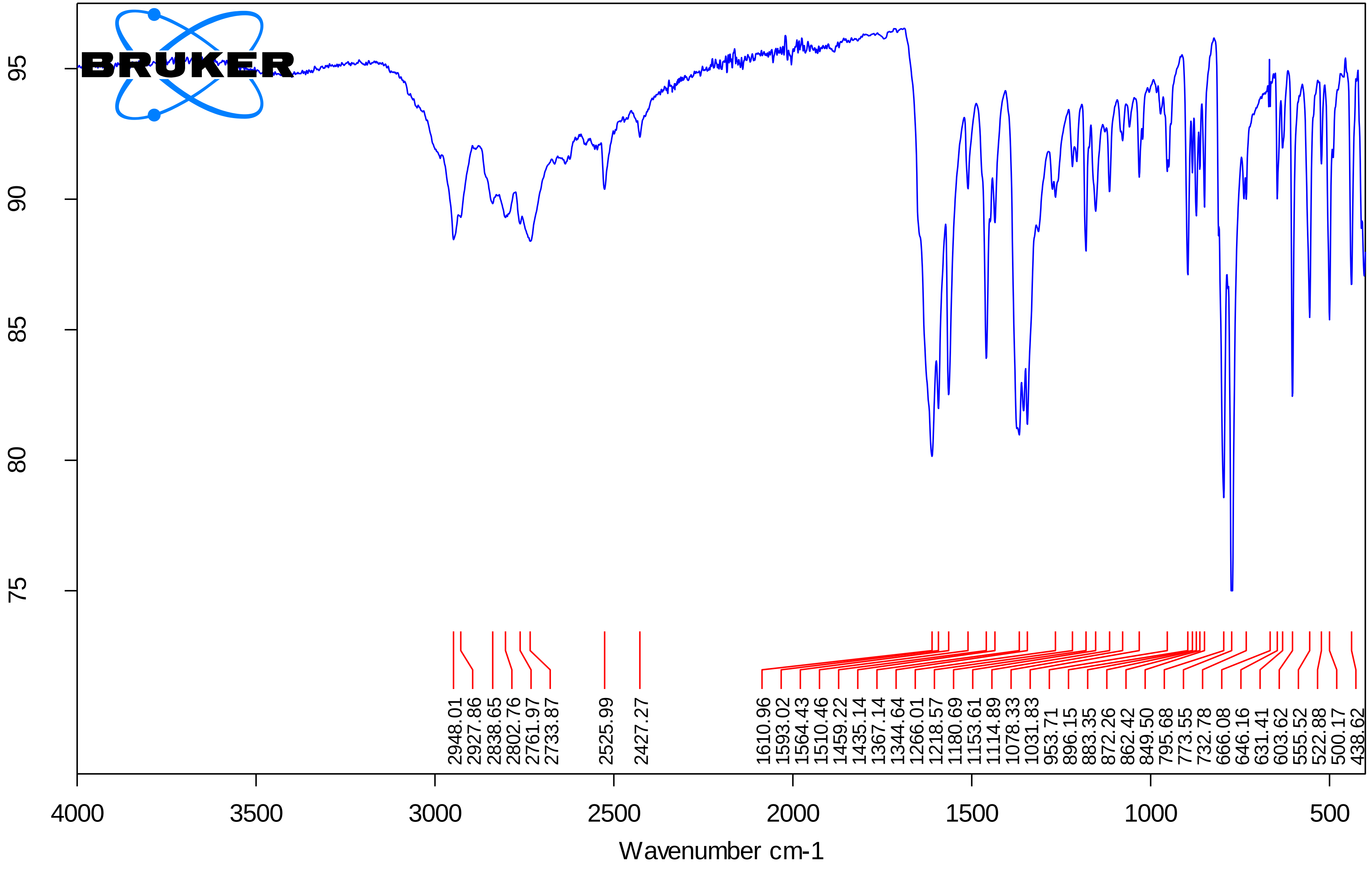

References

S1. Haendler, H.M. Copper quinaldinate monohydrate [aquabis(2-quinolinecarboxylato)copper(II)]; pentacoordinate copper. *Acta Crystallogr., Sect. C* **1986**, *42*, 147-149, doi:10.1107/S0108270186096981.

S2. Janiak, C. A critical account on π–π stacking in metal complexes with aromatic nitrogen-containing ligands. *J. Chem. Soc., Dalton Trans.* **2000**, 3885-3896, doi:10.1039/B003010O.

S3. Macrae, C.F.; Bruno, I.J.; Chisholm, J.A.; Edgington, P.R.; McCabe, P.; Pidcock, E.; Rodriguez-Monge, L.; Taylor, R.; van de Streek, J.; Wood, P.A. *Mercury CSD 2.0* - new features for the visualization and investigation of crystal structures. *J. Appl. Crystallogr.* **2008**, *41*, 466-470, doi:10.1107/S0021889807067908.

S4. Etter, M.C.; MacDonald, J.C.; Bernstein, J. Graph-set analysis of hydrogen-bond patterns in organic crystals. *Acta Crystallogr., Sect. B* **1990**, *46*, 256-262, doi:10.1107/S0108768189012929.
