# Supplementary material for: Beyond the Simple Copper(II) Coordination Chemistry with Quinaldinate and Secondary Amines
Source: Molecules. 2020 Mar 30;25(7):1573. doi: 10.3390/molecules25071573 (PMC7180772; doi:10.3390/molecules25071573)

checkCIF/PLATON report

You have not supplied any structure factors. As a result the full set of tests cannot be run.

THIS REPORT IS FOR GUIDANCE ONLY. IF USED AS PART OF A REVIEW PROCEDURE FOR PUBLICATION, IT SHOULD NOT REPLACE THE EXPERTISE OF AN EXPERIENCED CRYSTALLOGRAPHIC REFEREE.

No syntax errors found.   CIF dictionary   Interpreting this report

Datablock: 6

```
Bond precision:  C-C = 0.0027 A         Wavelength=0.71073

Cell:           a=13.6876(7)    b=10.0952(4)       c=16.4802(9)
                alpha=90        beta=106.995(5)    gamma=90
Temperature:    150 K

                Calculated                 Reported
Volume          2177.77(19)                2177.77(19)
Space group     P 21/c                     P 1 21/c 1
Hall group      -P 2ybc                    -P 2ybc
Moiety formula  C25 H23 Cu N3 O4           C25 H23 Cu N3 O4
Sum formula     C25 H23 Cu N3 O4           C25 H23 Cu N3 O4
Mr              493.01                     493.00
Dx,g cm-3       1.504                      1.504
Z               4                          4
Mu (mm-1)       1.042                      1.042
F000            1020.0                     1020.0
F000'           1021.65
h,k,lmax        19,14,23                   19,13,23
Nref            6672                       5898
Tmin,Tmax       0.477,0.732                0.568,1.000
Tmin'           0.430

Correction method= # Reported T Limits: Tmin=0.568 Tmax=1.000
AbsCorr = MULTI-SCAN

Data completeness= 0.884          Theta(max)= 30.520

R(reflections)= 0.0332( 5007)     wR2(reflections)= 0.0901( 5898)

S = 1.047                  Npar= 302
```

The following ALERTS were generated. Each ALERT has the format
test-name_ALERT_alert-type_alert-level.
Click on the hyperlinks for more details of the test.

Alert level G

```
PLAT063_ALERT_4_G Crystal Size Possibly too Large for Beam Size ..       0.80 mm
PLAT232_ALERT_2_G Hirshfeld Test Diff (M-X)  Cu1      --O11      .        5.5 s.u.
PLAT794_ALERT_5_G Tentative Bond Valency for Cu1       (II)      .       2.12 Info
PLAT883_ALERT_1_G No Info/Value for _atom_sites_solution_primary .     Please Do !
```

```
   0 ALERT level A = Most likely a serious problem - resolve or explain
   0 ALERT level B = A potentially serious problem, consider carefully
   0 ALERT level C = Check. Ensure it is not caused by an omission or oversight
   4 ALERT level G = General information/check it is not something unexpected

   1 ALERT type 1 CIF construction/syntax error, inconsistent or missing data
   1 ALERT type 2 Indicator that the structure model may be wrong or deficient
   0 ALERT type 3 Indicator that the structure quality may be low
   1 ALERT type 4 Improvement, methodology, query or suggestion
   1 ALERT type 5 Informative message, check
```

It is advisable to attempt to resolve as many as possible of the alerts in all categories. Often the minor alerts point to easily fixed oversights, errors and omissions in your CIF or refinement strategy, so attention to these fine details can be worthwhile. In order to resolve some of the more serious problems it may be necessary to carry out additional measurements or structure refinements. However, the purpose of your study may justify the reported deviations and the more serious of these should normally be commented upon in the discussion or experimental section of a paper or in the "special_details" fields of the CIF. checkCIF was carefully designed to identify outliers and unusual parameters, but every test has its limitations and alerts that are not important in a particular case may appear. Conversely, the absence of alerts does not guarantee there are no aspects of the results needing attention. It is up to the individual to critically assess their own results and, if necessary, seek expert advice.

Publication of your CIF in IUCr journals

A basic structural check has been run on your CIF. These basic checks will be run on all CIFs submitted for publication in IUCr journals (*Acta Crystallographica*, *Journal of Applied Crystallography*, *Journal of Synchrotron Radiation*); however, if you intend to submit to *Acta Crystallographica Section C* or *E* or *IUCrData*, you should make sure that full publication checks are run on the final version of your CIF prior to submission.

Publication of your CIF in other journals

Please refer to the *Notes for Authors* of the relevant journal for any special instructions relating to CIF submission.

PLATON version of 22/12/2019; check.def file version of 13/12/2019

Datablock 6 - ellipsoid plot

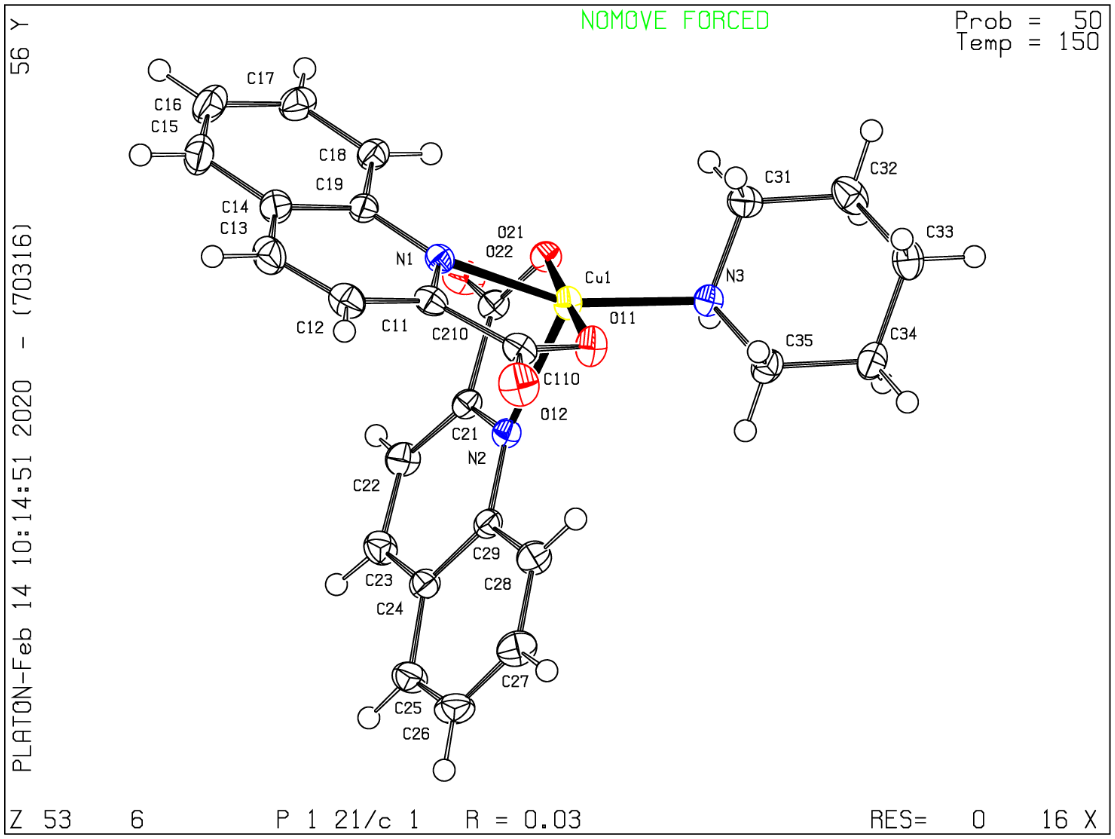

Supplement: Supplementary file 1 [file molecules-25-01573-s001.zip › checkcif for 6.pdf]
