# Supplementary material for: Beyond the Simple Copper(II) Coordination Chemistry with Quinaldinate and Secondary Amines
Source: Molecules. 2020 Mar 30;25(7):1573. doi: 10.3390/molecules25071573 (PMC7180772; doi:10.3390/molecules25071573)

# checkCIF/PLATON report

You have not supplied any structure factors. As a result the full set of tests cannot be run.

THIS REPORT IS FOR GUIDANCE ONLY. IF USED AS PART OF A REVIEW PROCEDURE FOR PUBLICATION, IT SHOULD NOT REPLACE THE EXPERTISE OF AN EXPERIENCED CRYSTALLOGRAPHIC REFEREE.

No syntax errors found. CIF dictionary Interpreting this report

## Datablock: 8

---

```
Bond precision:  C-C = 0.0024 A          Wavelength=1.54184

Cell:            a=11.8229(2)     b=7.6056(1)        c=16.2266(3)
                 alpha=90         beta=90.451(2)     gamma=90
Temperature:     150 K

                Calculated                Reported
Volume          1459.06(4)                1459.05(4)
Space group     P 21/n                    P 1 21/n 1
Hall group      -P 2yn                    -P 2yn
Moiety formula  C30 H34 Cu N4 O4          C30 H34 Cu N4 O4
Sum formula     C30 H34 Cu N4 O4          C30 H34 Cu N4 O4
Mr              578.16                    578.15
Dx,g cm-3       1.316                     1.316
Z               2                         2
Mu (mm-1)       1.384                     1.384
F000            606.0                     606.0
F000’           603.68
h,k,lmax        14,9,20                   14,9,20
Nref            3011                      2937
Tmin,Tmax       0.847,0.933               0.505,1.000
Tmin’           0.575

Correction method= # Reported T Limits: Tmin=0.505 Tmax=1.000
AbsCorr = MULTI-SCAN

Data completeness= 0.975          Theta(max)= 74.994

R(reflections)= 0.0356( 2749)     wR2(reflections)= 0.0951( 2937)

S = 1.060                  Npar= 182
```

---

The following ALERTS were generated. Each ALERT has the format
**test-name_ALERT_alert-type_alert-level**.
Click on the hyperlinks for more details of the test.

### Alert level B

```
PLAT601_ALERT_2_B Structure Contains Solvent Accessible VOIDS of .        102 Ang**3
```

### Alert level C

```
PLAT369_ALERT_2_C Long   C(sp2)-C(sp2) Bond  C11      - C110     .       1.53 Ang.
```

### Alert level G

```
PLAT013_ALERT_1_G N.O.K.   _shelx_hkl_checksum Found in CIF ......     Please Check
PLAT794_ALERT_5_G Tentative Bond Valency for Cu1       (II)      .       2.03 Info
PLAT883_ALERT_1_G No Info/Value for _atom_sites_solution_primary .     Please Do !
```

```
   0 ALERT level A = Most likely a serious problem - resolve or explain
   1 ALERT level B = A potentially serious problem, consider carefully
   1 ALERT level C = Check. Ensure it is not caused by an omission or oversight
   3 ALERT level G = General information/check it is not something unexpected

   2 ALERT type 1 CIF construction/syntax error, inconsistent or missing data
   2 ALERT type 2 Indicator that the structure model may be wrong or deficient
   0 ALERT type 3 Indicator that the structure quality may be low
   0 ALERT type 4 Improvement, methodology, query or suggestion
   1 ALERT type 5 Informative message, check
```

It is advisable to attempt to resolve as many as possible of the alerts in all categories. Often the minor alerts point to easily fixed oversights, errors and omissions in your CIF or refinement strategy, so attention to these fine details can be worthwhile. In order to resolve some of the more serious problems it may be necessary to carry out additional measurements or structure refinements. However, the purpose of your study may justify the reported deviations and the more serious of these should normally be commented upon in the discussion or experimental section of a paper or in the "special_details" fields of the CIF. checkCIF was carefully designed to identify outliers and unusual parameters, but every test has its limitations and alerts that are not important in a particular case may appear. Conversely, the absence of alerts does not guarantee there are no aspects of the results needing attention. It is up to the individual to critically assess their own results and, if necessary, seek expert advice.

**Publication of your CIF in IUCr journals**

A basic structural check has been run on your CIF. These basic checks will be run on all CIFs submitted for publication in IUCr journals (*Acta Crystallographica*, *Journal of Applied Crystallography*, *Journal of Synchrotron Radiation*); however, if you intend to submit to *Acta Crystallographica Section C* or *E* or *IUCrData*, you should make sure that full publication checks are run on the final version of your CIF prior to submission.

**Publication of your CIF in other journals**

Please refer to the *Notes for Authors* of the relevant journal for any special instructions relating to CIF submission.

**PLATON version of 22/12/2019; check.def file version of 13/12/2019**

**Datablock 8** - ellipsoid plot

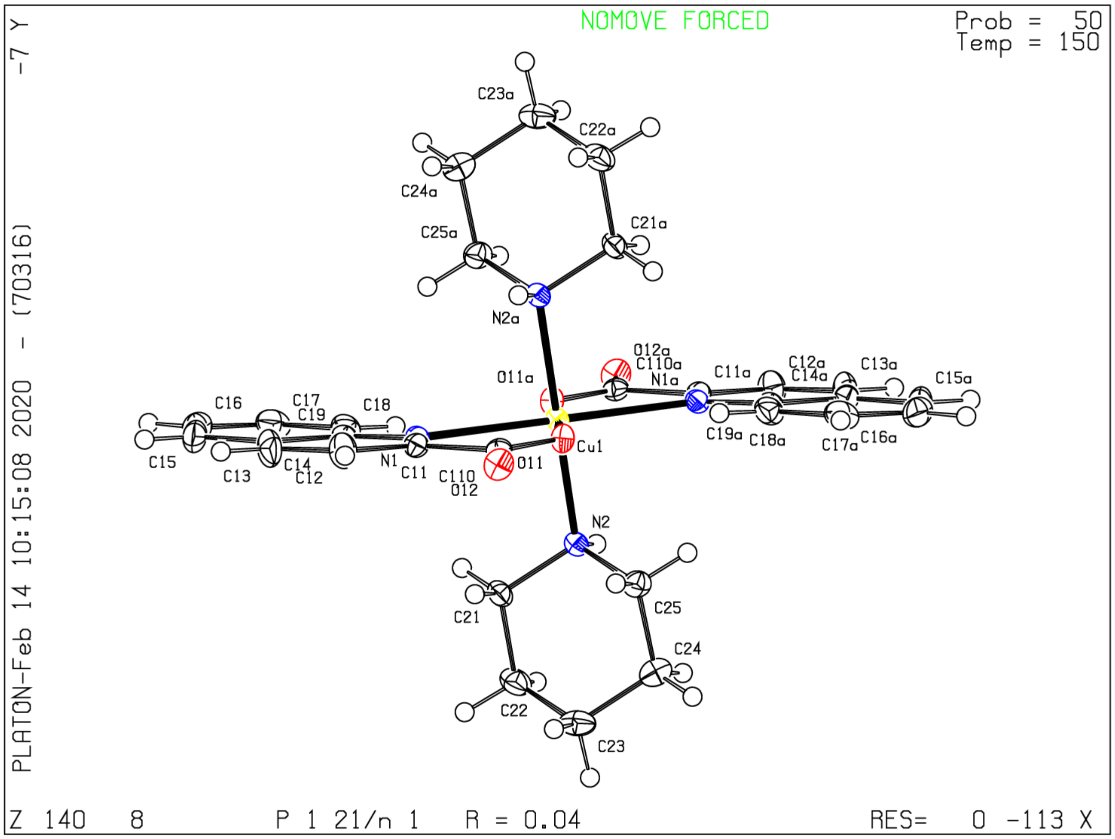

Supplement: Supplementary file 1 [file molecules-25-01573-s001.zip › checkcif for 8.pdf]
